# A dispensable SepIVA orthologue in *Streptomyces venezuelae* is associated with polar growth and not cell division

**DOI:** 10.1186/s12866-024-03625-6

**Published:** 2024-11-18

**Authors:** Beer Chakra Sen, Parminder Singh Mavi, Oihane Irazoki, Susmita Datta, Sebastian Kaiser, Felipe Cava, Klas Flärdh

**Affiliations:** 1 Department of Biology, Lund University, Kontaktvägen 13, Lund, 223 62 Sweden; 2 Department of Molecular Biology and Laboratory for Molecular Infection Medicine Sweden, Umeå Centre for Microbial Research, SciLifeLab, Umeå University, Umeå, Sweden

**Keywords:** Cell division, Cell wall synthesis, FtsZ, Polar growth, *Streptomyces*

## Abstract

**Background:**

SepIVA has been reported to be an essential septation factor in *Mycolicibacterium smegmatis* and *Mycobacterium tuberculosis*. It is a coiled-coil protein with similarity to DivIVA, a protein necessary for polar growth in members of the phylum Actinomycetota. Orthologues of SepIVA are broadly distributed among actinomycetes, including in *Streptomyces* spp.

**Results:**

To clarify the role of SepIVA and its potential involvement in cell division in streptomycetes, we generated *sepIVA* deletion mutants in *Streptomyces venezuelae* and found that *sepIVA* is dispensable for growth, cell division and sporulation. Further, mNeonGreen-SepIVA fusion protein did not localize at division septa, and we found no evidence of involvement of SepIVA in cell division. Instead, mNeonGreen-SepIVA was accumulated at the tips of growing vegetative hyphae in ways reminiscent of the apical localization of polarisome components like DivIVA. Bacterial two-hybrid system analyses revealed an interaction between SepIVA and DivIVA. The results indicate that SepIVA is associated with polar growth. However, no phenotypic effects of *sepIVA* deletion could be detected, and no evidence was observed of redundancy with the other DivIVA-like coiled-coil proteins Scy and FilP that are also associated with apical growth in streptomycetes.

**Conclusions:**

We conclude that *S. venezuelae* SepIVA, in contrast to the situation in mycobacteria, is dispensable for growth and viability. The results suggest that it is associated with polar growth rather than septum formation.

**Supplementary Information:**

The online version contains supplementary material available at 10.1186/s12866-024-03625-6.

## Background

The peptidoglycan cell wall sacculus provides protection of the bacterial cytoplasm and maintains cell shape. For bacteria to grow and proliferate, the sacculus has to be enlarged, remodelled and divided in a highly controlled fashion. Peptidoglycan synthases and hydrolases are organized in large protein complexes and directed to be active at the right times and places in the cell. Cytoskeletal elements have key roles in organizing cell wall synthesis. FtsZ orchestrates cell division by directing the divisome complex at cell division sites, while MreB proteins organize cell elongation in rod-shaped bacteria by directing elongasome complexes to act along the lateral wall of the cell (reviewed in e.g [1–3]). Members of the phylum Actinomycetota accomplish cell elongation without employing MreB proteins. They grow as rods or filamentous cells by building the cell wall sacculus at the cell poles, and this polar mode of growth is dependent on the cell polarity determinant protein DivIVA [4–9]. DivIVA is found in many bacterial groups, mainly Gram-positives, and is characterized by an N-terminal lipid-binding domain and a C-terminal oligomerization domain, both dominated by coiled-coil structure [10–13]. In cells, DivIVA shows preference for curved membranes and assembles at cell poles and in many organisms also at septation sites [14, 15]. A range of roles have been described in different bacteria, typically involving recruitment of proteins to cell poles or late stages of cell division (for review, see [12]).

In Actinomycetota, *divIVA* orthologues are essential for polar growth. In mycobacteria, DivIVA (also referred to as Wag31) is localized to cell poles and septation sites and is required for cell elongation [6, 8, 9, 16]. It appears to focus cell wall synthesis activity to the cell poles, affects the stability of the intracellular membrane domain (IMD) that is associated with polar cell envelope biogenesis, and has been reported to interact with cell wall synthesis-related proteins [6, 17–21]. In *Corynebacterium glutamicum*, DivIVA localizes to cell poles and is required for rod shape and polar growth [7, 22], and it interacts with and recruits the peptidoglycan transglycosylase RodA to cell poles [23, 24]. Similarly, the filamentous and mycelial mode of growth of streptomycetes depends on clusters of DivIVA at hyphal tips [4], and these clusters, referred to as polarisomes, are also instrumental in the *de novo* establishment of growth zones that occur during the formation of lateral branches [5, 25].

Two additional coiled-coil proteins, Scy and FilP, are targeted to hyphal tips in streptomycetes, contribute to control of cell shape, and interact with DivIVA [26–29]. Both FilP and Scy belong to a large group of actinomycete coiled-coil proteins that share a conserved N-terminal motif similar to DivIVA, referred to as a DivIVA-like domain, and appear to have evolved from DivIVA [30].

An additional coiled-coil protein (SepIVA) with a DivIVA-like domain has been identified as an interaction partner of cell division protein FtsQ in *Mycolicibacterium smegmatis* [31, 32]. SepIVA localizes to cell division septa in mycobacteria and is recruited to these sites at a late stage of cell division. The protein has also been observed at what appeared to correspond to the sub-apical IMD domains, suggesting possible involvement in both cell division and cell wall synthesis [32]. SepIVA is reported to be essential for viability in *M. smegmatis* and *Mycobacterium tuberculosis* [31–34]. However, viable *sepIVA* mutants of *M. smegmatis* were recently described that show slow growth and perturbed cell morphology, suggesting that *sepIVA* has an important role in mycobacterial growth and division but may not always be essential [35]. Homologues of *sepIVA* were found among various members of the Corynebacterineae suborder, and also mentioned to be present in other actinomycetes, including streptomycetes [35].

Streptomycetes have a complex developmental life cycle. In the vegetative stage, filamentous hyphae extend by polar cell wall growth and create new growth zones by lateral branching [36, 37]. Upon nutrient depletion and other stimuli, a developmental program is initiated leading to the formation of a sporulating aerial mycelium on the surface of colonies [38, 39]. Sporulation involves synchronous formation of multiple sporulation septa along apical parts of aerial hyphae. Septation is coordinated with segregation of single chromosomes into prespore compartments and followed by maturation of the dormant spores. The essential role of *sepIVA* in mycobacteria and its involvement in cell division and possibly cell wall assembly [31, 32, 35, 40] raises the question of its function in streptomycetes. However, the complex ways by which these organisms coordinate growth, morphological differentiation and cell division makes it hard to predict how the findings reported for mycobacterial *sepIVA* would translate to the filamentous streptomycetes. As there are at least two distinct types of cell divisions, both hyphal crosswalls in vegetative hyphae that surprisingly are dispensable for viability [41, 42], and sporulation septa that are required for spore formation [43–46], it is not obvious what stage of the life cycle could be affected by *sepIVA*. We have here investigated the role of *sepIVA* in the model organism *Streptomyces venezuelae*. We show that *sepIVA* orthologues are found in all suborders of the Actinomycetales. However, the function is not conserved between mycobacteria and streptomycetes, and we see no indication of an involvement of *sepIVA* in cell division and no localization to cell division sites at any stage of the developmental life cycle of *S. venezuelae*. In contrast, using genetic and cell biological approaches, we find that SepIVA is dispensable for growth and provide evidence that it is associated with polar growth and hyphal tip extension in *S. venezuelae*.

## Methods

### Bacterial strains, plasmids, oligonucleotides, and growth conditions

Bacterial strains and plasmids used in this study are listed in Table 1 and Supplementary Table S1, respectively. The *S. venezuelae* strains are derivatives of strain NRRL B-65442. *E. coli* strain DH5α was used for cloning, while strain DY380 was used to carry out λRed-mediated mutagenesis of cosmids. *E. coli* strain ET12567/pUZ8002 was used for mobilization of *oriT*-containing cosmids and plasmids into *S. venezuelae* as described previously [47]. Media, growth conditions and genetic manipulation were generally performed as described previously for *E. coli* [48] and *Streptomyces* strains [49], unless otherwise stated. *E. coli* strains were grown in lysogeny broth (LB) or on LB agar [50] with 10 g l^− 1^ NaCl, or without NaCl when hygromycin was used for selection. *S. venezuelae* cells were grown in maltose-yeast extract-malt extract medium (MYM) or MYM agar, as described by Bush et al. [47]. Chitin agar and MOPS glucose minimal agar were prepared as previously described [51, 52]. For monitoring of exploratory growth phenotype, cultures were grown on yeast extract-peptone (YP) agar medium as described previously [53]. Antibiotics were used with the following final concentrations: 50 µg ml^− 1^ apramycin, 100 µg ml^− 1^ carbenicillin, 25 µg ml^− 1^ chloramphenicol, 25 µg ml^− 1^ hygromycin, 50 µg ml^− 1^ kanamycin, and 20 µg ml^–1^ nalidixic acid. Oligonucleotides used in the study are listed in Supplementary Table S2.

**Table 1:** Bacterial strains used in this study

Strains	Genotype or Relevant characteristics	Reference or source
**Escherichia coli**		
BTH101	*F* ^*–*^ *cya-99 araD139 galE15 galK16 rpsL1 (Str* ^*r*^ *) hsdR2 mcrA1 mcrB1*	[54]
DH5α	*supE44* Δ*lacU169* (*Φ80 lac* ZΔ*M15*) *hsdR17 recA1 endA1 gyrA96 thi*-1 *relA1*	[55]
DY380	F- *mcrA* Δ(*mrr-hsdRMS*-*mcrBC*) Φ80d *lacZM15 ΔlacX74 deoR recA1 endA1 araD139* Δ(*ara*, *leu*)*7649 galU galK rspL nupG* [ *λcI857* (*cro-bioA*) < > *tet*]	[56]
ET12567/pUZ8002	*dam*-13::Tn *9 dcm*-6 *hsdM*, carrying helper plasmid pUZ8002	[49]
***Streptomyces venezuelae***		
NRRL B-65442	Wild type *S. venezuelae* strain	[57]
LUV041	*attB*_*ΦBT1*_::pMS82	[58]
LUV052	*attB*_*ΦBT1*_::pKF543(*ftsZ-ypet*)	[58]
LUV080	*ΔsepIVA*::*apra*^1^	This work
LUV112	*ΔsepIVA::apra attB*_*ΦBT1*_::pKF652(*sepIVA*)	This work
LUV119	*ΔsepIVA::apra attB*_*ΦBT1*_::pMS82	This work
LUV125	*ΔsepIVA::apra attB*_*ΦBT1*_::pKF543(*ftsZ-ypet*)	This work
LUV169	*attB*_*ΦBT1*_::pKF703(kasOp*-mNeongreen-sepIVA)	This work
LUV171	*attB*_*ΦBT1*_::pSS76(kasOp*)	This work
LUV189	*attB*_*ΦBT1*_::pKF733(kasOp*-mNeongreen-sepIVA divIVA-mCherry)	
LUV339	*ΔsepIVA::apra Δ(scy-filP)::FRT*	This work
LUV340	*ΔsepIVA*::*apra*	This work
NA1256	*Δ(scy-filP)::FRT*	[27]

^1^*apra* denotes here a cassette containing both the apramycin resistance gene *aac(3)IV* and an *oriT*, derived from plasmid pIJ773

### Isolation of *sepIVA* mutant strain

**Fig. 1:**
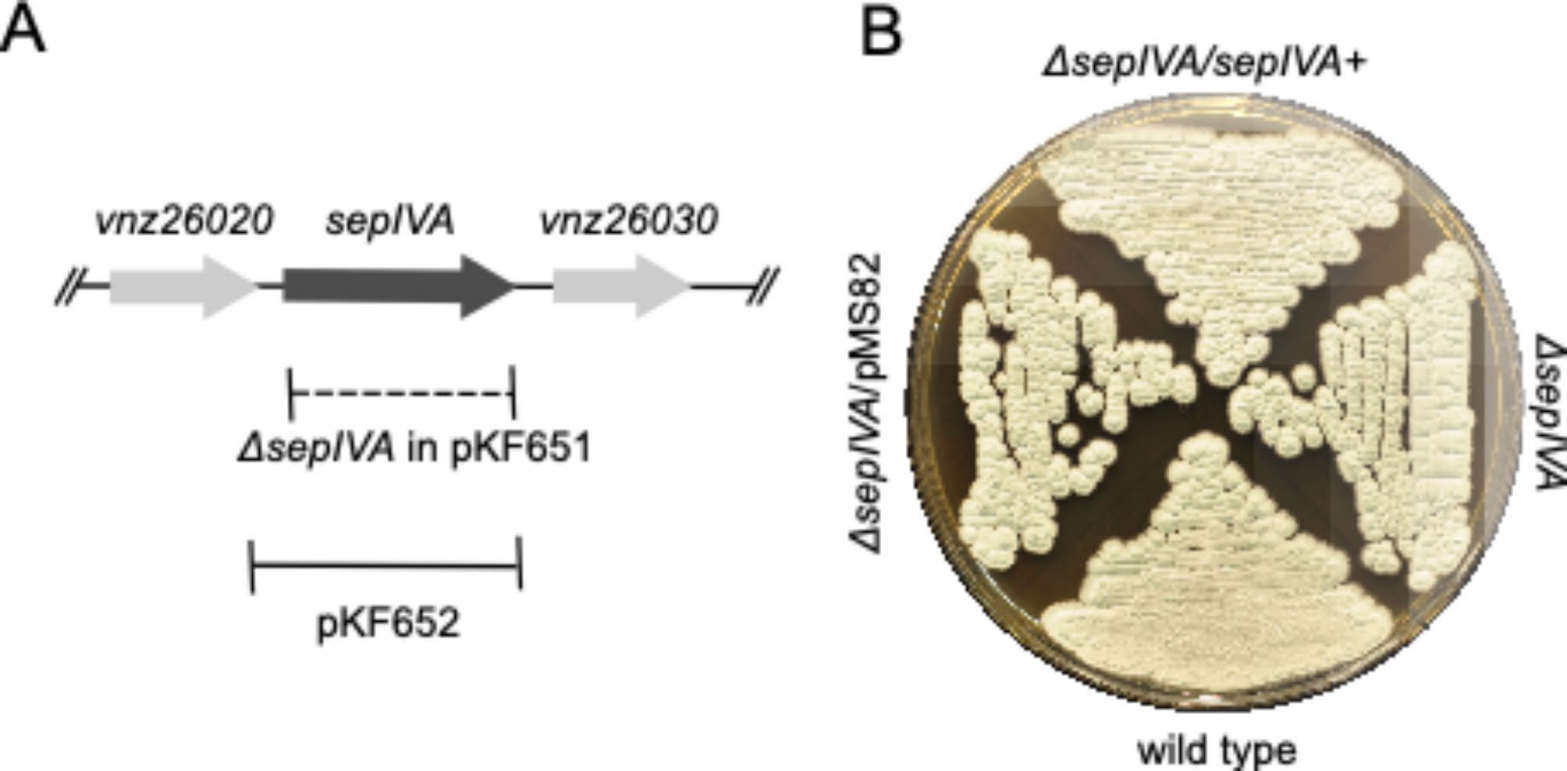
Construction and colony phenotype of *S. venezuelae sepIVA* null mutants. **A**) Schematic map of *sepIVA* locus, showing also the limits of the *ΔsepIVA::apra* gene replacement in the *sepIVA* null mutants. For complementation, plasmid pKF652 was used and the region included is indicated (solid line). **B**) Colony appearance of *sepIVA* mutant together with complemented and wild-type strains on MYM agar plate grown for 4–5 days at 30 °C. The *S. venezuelae* strains are LUV080 (*ΔsepIVA*); LUV112 (*ΔsepIVA attB*_*ΦBT1*_::pKF652[*sepIVA*]); LUV119 (*ΔsepIVA attB*_*ΦBT1*_::pMS82); and wild-type parent strain NRRL B-65442

*S. venezuelae sepIVA* deletion strain was constructed essentially following the ‘Redirect’ PCR targeting protocol [59], but employing a different strain and protocol for λRed recombineering of cosmid DNA in *E. coli* [60]. The apramycin resistance cassette from pIJ773 ([*aac(3)IV-oriT*], hereafter referred to as *apra*) was amplified using primer pair KF1568/KF1569 and introduced into *E. coli* strain DY380 harboring cosmid 3-B07 containing *sepIVA* (*vnz26025*). The mutagenized cosmid, named pKF651, was verified for the replacement of *sepIVA* gene with *apra* resistance cassette by PCR with primer pairs KF1570/KF435 and KF1570/KF1571. The verified pKF651 was transferred to *S. venezuelae* by conjugation, as described previously [49] with some modifications. Conjugation mix containing *E. coli* donor and *S. venezuelae* recipient cells was plated on Soya Flour-Mannitol (SFM) agar supplemented with 10 mM MgCl_2_ and was incubated overnight (15–16 h) at room temperature. Thereafter, the plates were overlaid with nalidixic acid (20 µg ml^–1^) and apramycin (50 µg ml^–1^) and were further incubated at 30 °C until ex-conjugants appeared (2–4 days). The ex-conjugants were purified by streaking on MYM agar containing nalidixic acid and apramycin to select for *S. venezuelae* recombinants with the *apra* cassette integrated into the chromosome. Putative null mutants were identified based on their apramycin resistance and kanamycin sensitivity. Genomic DNA from mutant candidates was isolated and PCR verification was done using primer pairs KF1570/KF1571 flanking the *sepIVA* locus and KF1570/KF435 detecting the gene replacement. Null mutants were readily isolated in which *sepIVA* had been replaced with the *apra* cassette. (Fig. 1A). Four *S. venezuelae sepIVA* null mutants were isolated and found to be phenotypically indistinguishable, both macroscopically and microscopically, when grown on MYM agar and in liquid MYM. One representative *sepIVA* mutant was named LUV080, and its genotype was confirmed by genome sequencing. Genomic DNA was prepared using NucleoSpin Plant II kit (Macherey-Nagel, Germany) according to manufacturer’s recommendations, after first treating mycelium with lysozyme to release protoplasts [49]. The quality and concentration of genomic DNA were assessed using gel-electrophoresis, NanoDrop spectrophotometer, and Qubit fluorometer (Thermo Fisher Scientific). Whole-genome sequencing was carried out on an Illumina platform to produce paired-end reads. Library preparation and sequencing were performed by Eurofins Genomics using the INVIEW Resequencing service. Sequencing reads were processed using tools in the Galaxy web platform and the public server at usegalaxy.org to analyze the data [61]. Paired-end reads were aligned to *S. venezuelae* wild-type NRRL B-65,442 reference genome (NCBI accession number NZ_CP018074.1) using bowtie2. Single nucleotide polymorphisms (SNPs) and small insertions/deletions (Indels) were identified using snippy and bcftools mpileup and bcftools call. In a subsequent experiment, the *ΔsepIVA*::*apra* gene replacement was introduced and verified in the *Δ(scy-filP)* mutant background to yield strain LUV339, and an additional *ΔsepIVA*::*apra* mutant was isolated in the wild-type background as a control in these experiments, strain LUV340.

### Construction of plasmids

For *in trans* complementation tests, the *sepIVA* gene was PCR amplified with primer KF1574 containing NdeI restriction site and primer KF1575 with KpnI restriction site. The amplified PCR product was cloned into NdeI and KpnI sites of plasmid pIJ10770, generating plasmid pKF652. The inserted *sepIVA* gene was verified by PCR using primer pair KF1272/KF1246 and by DNA sequencing. The plasmid was introduced into *S. venezuelae* strains by conjugation and integrated at the фBT1 attachment site.

For the study of subcellular localization of SepIVA, PCR amplification of *sepIVA* was done using primers KF1646/KF1647 with AflII and HindIII restriction sites. The PCR product was digested and ligated into pKF699 (*kasO*p*-mNeongreen-cvnD2) resulting in the plasmid pKF703 (*kasO*p*-mNeongreen-sepIVA). Thus, the fusion construct is expressed from a relatively strong constitutive promoter [62]. Verification of pKF703 was done by restriction mapping and DNA sequencing using primer pairs KF1646/KF1647. Plasmid pKF703 was conjugated into *S. venezuelae* wild type as described earlier, resulting in the strain LUV169. The *kasO*p*-mNeongreen-sepIVA fragment was also excised using XbaI and AvrII and ligated in the SpeI site of pSS204, resulting in plasmid pKF733, which allows studies of co-localisation of mNeongreen-SepIVA and DivIVA-mCherry.

In order to construct plasmids for bacterial two-hybrid assay [54], *sepIVA* was PCR amplified using primer pairs KF1656/KF1657 and *S. venezuelae* genomic DNA as a template. The PCR product was digested using XbaI and KpnI and ligated into pUT18, pKT25 and pKNT25. The resulting plasmids were named pKF705, pKF707 and pKF708, respectively. The *divIVA* plasmids were created by amplifying *divIVA* from chromosomal DNA of *S. venezuelae* with primers KF1370/KF1371, digesting PCR products with XbaI and KpnI and ligating to the corresponding sites in the BACTH vectors. Plasmid inserts were verified by DNA sequencing.

For creating pKF756, *sepIVA* was amplified with KF1646 and KF1579, digested with AflII and XhoI and ligated with similarly digested pKF748 to create kasOp* driven expression of *sepIVA*. pKF748 was derived from pSS76 by overlapping fragments with partial FLAG-tag sequence (created from pSS76 as template with primer sets KF1727/KF1752 and KF1753/KF1751), digesting pSS76 and the overlapping product with KpnI and HindIII and ligation. All plasmid constructs were verified by sequencing DNA inserts by Sanger sequencing at Eurofins Genomics.

### Microscopy

For phase-contrast microscopy of aerial hyphae and spores, colonies were grown at 30 °C on MYM agar for 4–5 days. Aerial hyphae and spores were sampled by pressing a cover slip against the colony surface and then mounting it on a slide coated with 1% agarose in phosphate-buffered saline (PBS). To observe growth during vegetative stage, spores were diluted and inoculated onto cellophane membranes placed on the top of MYM agar or MOPS glucose minimal agar. Plates were incubated at 30 °C for 17 h and membranes were carefully transferred to agarose-coated (1% in PBS) slides. Prepared slides were imaged using phase-contrast microscopy. Cell wall and nucleoid staining of cultures with Wheat germ agglutinin-Oregon Green (WGA-Oregon Green; Molecular Probes) and 7-Aminoactinomycin D (7-AAD; Molecular Probes) was done as described previously [63]. To observe subcellular localization of fusion proteins by fluorescence microscopy, cells were grown in liquid MYM and transferred to agarose-coated slides. To study FtsZ dynamics and SepIVA localization by time-lapse imaging, bacteria were cultivated in the CellASIC ONIX2 microfluidic system and B04A-03 microfluidic plates (Merck Millipore), as described previously [64]. Imaging was performed on a Zeiss AxioObserver.Z1 microscope with Illuminator HXP 120 V lamp (Zeiss), appropriate fluorescence filters, Zeiss Plan-Apochromat 63×/1.4 Oil Ph3 or 100×/1.4 Oil Ph3 objective, ZEN software (Zeiss), and an ORCA Flash 4.0 LT camera (Hamamatsu). ImageJ/Fiji [65] was used to generate images and movies, as previously described [64]. Fluorescence profiles for SepIVA localization were obtained using ZEN and a width of 5 pixels. Prior to analysis, profiles were aligned at the hyphal apex guided by intensity profiles from the phase contrast channel.

### Bacterial two-hybrid assay

The ‘T25’ and ‘T18’ fusion plasmids were used to co-transform chemically competent *E. coli* BTH101 cells. Transformants were selected on LB agar containing carbenicillin and kanamycin. Detection of β-galactosidase activity on agar plates or in microtiter trays was done essentially as recommended previously [66]. Three individual transformants per combination were grown overnight at 30 °C in LB with antibiotics and 0.5 mM isopropyl β-D-1-thiogalactopyranoside (IPTG). The resulting cultures were spotted (3 µl) onto LB agar with 40 µg ml^− 1^ X-gal) and 0.5 mM IPTG and appropriate antibiotics. Plates were incubated at 30 °C in dark conditions for 24–48 h before photographs were taken. For quantitative assays, three co-transformants were grown overnight in liquid LB with ampicillin, kanamycin and IPTG at 30 °C. OD_600_ was measured in a microplate reader on the next day. β-galactosidase assays were done as described [66] and OD_405_ was measured every 2 min in a FLUOstar OPTIMA Microplate Reader (BMG Labtech).

### Analyses of peptidoglycan and cell wall structure

Cultures were grown in liquid MYM until OD_600_ reached 1.0. Mycelium was harvested by centrifugation and washed 3 times in PBS at 4 °C. Peptidoglycan (PG) samples were analysed following the 24 h protocol as described previously, with some modifications [67, 68]. In brief, samples were boiled in SDS 5% for 2 h and sacculi were repeatedly washed with MilliQ water by ultracentrifugation (541.000 × g, 10 min, 20ºC), repeatedly washed, sonicated, and treated with α-amylase, DNase, RNase, and trypsin, and enzymes were inactivated by boiling. Wall teichoic acids were removed with 1 M HCl. The samples were finally treated with muramidase (100 µg ml^− 1^) for 15 h at 37ºC. Muramidase digestion was stopped by boiling and, coagulated proteins were removed by centrifugation (10 min, 18.800 × g). The supernatants were first adjusted to pH 8.5-9.0 with sodium borate buffer and then sodium borohydride was added to a final concentration of 10 mg ml^− 1^. After reduction for 30 min at room temperature, the pH was adjusted to pH 3.5 with orthophosphoric acid.

UPLC analyses of muropeptides were performed on a Waters UPLC system (Waters Corporation, USA) equipped with an ACQUITY UPLC BEH C18 Column, 130Å, 1.7 μm, 2.1 mm X 150 mm (Waters, USA) and a dual wavelength absorbance detector. Elution of muropeptides was detected at 204 nm. Muropeptides were separated at 45ºC using a linear gradient from buffer A (formic acid 0.1% in water) to buffer B (formic acid 0.1% in acetonitrile) in a 30-minute run, under a 0.50 ml min^− 1^ flow.

Relative total PG amounts were calculated by comparison of the total intensities of the chromatograms (total area) from three biological replicas normalized to the same initial biomass and extracted with the same volumes. Quantification of muropeptides was based on their relative abundances (relative area of the corresponding peak) normalized to their molar ratio.

For analyses of overall appearance and thickness of cell walls in vegetatively growing hyphae, cultures were grown in liquid culture in MYM medium for 12 h. Hyphae were harvested by centrifugation. The supernatant was removed and replaced with freshly prepared 3% glutaraldehyde in 0.1 M sodium cacodylate buffer (pH 7.4) and incubated for 12 h at 7 °C. After fixation the fixative solution was removed, and the pellets were washed with 0.1 M sodium cacodylate buffer (pH 7.4). The pellets were postfixed in 2% osmium tetroxide in distilled water at 7 °C for 1 h. The specimens were then dehydrated in a graded ethanol series (70% 2 × 10 min, 96% 2 × 10 min, 100% 2 × 15 min) and embedded in Pelco Eponate 12 resin (Ted Pella) via acetone. Ultrathin Sect. (50 nm) were cut with a Leica UC7 with a diamond knife. The sections were stained with uranyl acetate (2%, 30 min) and Reynolds lead citrate (3 min) [69], mounted on copper grids, and viewed with a JEOL 1400 Plus Transmission Electron Microscope at 100 kV. The thickness of the peptidoglycan cell wall layer was measured at multiple points in hyphae that appeared to have been cross-sectioned perpendicularly to the hyphal length axis.

GraphPad PRISM Software (Inc., San Diego CA, www.graphpad.com) was used for statistical analysis.

### SepIVA orthologues and structure models

To find putative orthologues of SepIVA in the different suborders of Actinomycetales, *M. tuberculosis* SepIVA was used to search for homologous proteins with BLAST in genomes of each Actinomycetales suborder in the NCBI Microbial Genomes resource (https://www.ncbi.nlm.nih.gov/genome/microbes/). A few representatives of putative SepIVA homologues were selected from each suborder, and were then subjected to analysis and comparison of the genomic context with the tool webFlaGs [70]. Proteins encoded by genes from an obviously different genomic context were removed and only those originating from a locus with conserved flanking genes similar to those of known *sepIVA* genes were kept.

Predictions of the structure of homodimers of specific proteins were performed using Alphafold multimer via the LU-fold facility at Lund University (https://www.medicine.lu.se/research-and-research-studies/house-infrastructure/list-research-infrastructures/lu-fold). Five models were obtained per sequence and ranked according to the iptm + ptm score and the pDockQ score [71, 72]. The highest ranking models are displayed.

## Results

### *sepIVA* is broadly conserved in actinomycetales

The presence of several genes for coiled-coil proteins with DivIVA-like domains complicates the identification of SepIVA orthologues in actinomycete genomes. To clarify how widespread the SepIVA orthologues are, we first searched with BLAST for potential SepIVA homologs in the different subgroups of the order Actinomycetales, using *M. tuberculosis* SepIVA as query and then considered genome context using the webFlaGs tool. In this way, likely orthologs of SepIVA were found to be encoded in similar genomic contexts in a wide range of organisms, including representatives from all the suborders of Actinomycetales (Supplementary Fig. S1B), thus showing a broad distribution of SepIVA among actinomycetes. In *S. venezuelae* NRRL B-65442, the gene *vnz26025* encodes a protein that upon alignment to SepIVA from *M. smegmatis* shows 32% identity and 52% similarity over 238 aligned residues (Supplementary Fig. S2A). In similarity to the corresponding gene in other *Streptomyces* genomes, *vnz26025* has several flanking genes that are similar to those flanking for example *M. smegmatis sepIVA* (Supplementary Fig. S1A). Thus, *vnz26025* (WP_150265703.1) will be named *sepIVA* and regarded as an orthologue of mycobacterial *sepIVA*. The *S. venezuelae* SepIVA is about 100 residues longer (total 350 aa) than the *M. smegmatis* counterpart due to a C-terminal extension of sequence predicted to be mainly disordered, but the N-terminal parts are predicted to fold into highly similar structures (Supplementary Fig. S2). Analysis of a publicly available global transcription start site mapping data set in *S. venezuelae* showed a likely transcription start point around 38 base pairs upstream of the annotated translation start and further suggested a low and constitutive level of expression throughout a liquid culture growth cycle (Supplementary Fig. S3).

### The *sepIVA* gene is dispensable in *S. venezuelae*

A *sepIVA* deletion mutant was generated by replacing the entire coding region with apramycin (*apra*) resistance cassette (Fig. 1A). The genotype of a representative mutant (LUV080) was confirmed by genome sequencing. In addition to the precise deletion of *sepIVA*, it has the exact same nine single nucleotide differences from the reference genome (NCBI accession number NZ_CP018074.1) as we see in our lab strain of *S. venezuelae* NRRL B-65442 (i.e. LUV080 differs from its parent strain only by lacking *sepIVA*). On MYM agar, the *sepIVA* mutant LUV080 had the same colony appearance and green-greyish pigmentation as the wild-type parent strain, suggesting that there is no strong defect in growth or sporulation (Fig. 1B). The mutant strain carrying a plasmid with the entire coding sequence and 200 bases of the intergenic region upstream of *sepIVA* (strain LUV112) did not deviate from the null mutant, the mutant carrying empty pMS82 vector, or the wild-type parent strain with respect to colony morphology and pigmentation on MYM agar plates (Fig. 1B). To test for medium-dependent phenotypes, a *sepIVA* mutant was grown on chitin agar and MOPS glucose minimal agar plates together with wild-type strain, revealing unaffected colony morphology and pigmentation in the mutant compared to the wild type (Supplementary Fig. S4).

### SepIVA does not affect cell division in *S. venezuelae*

**Fig. 2:**
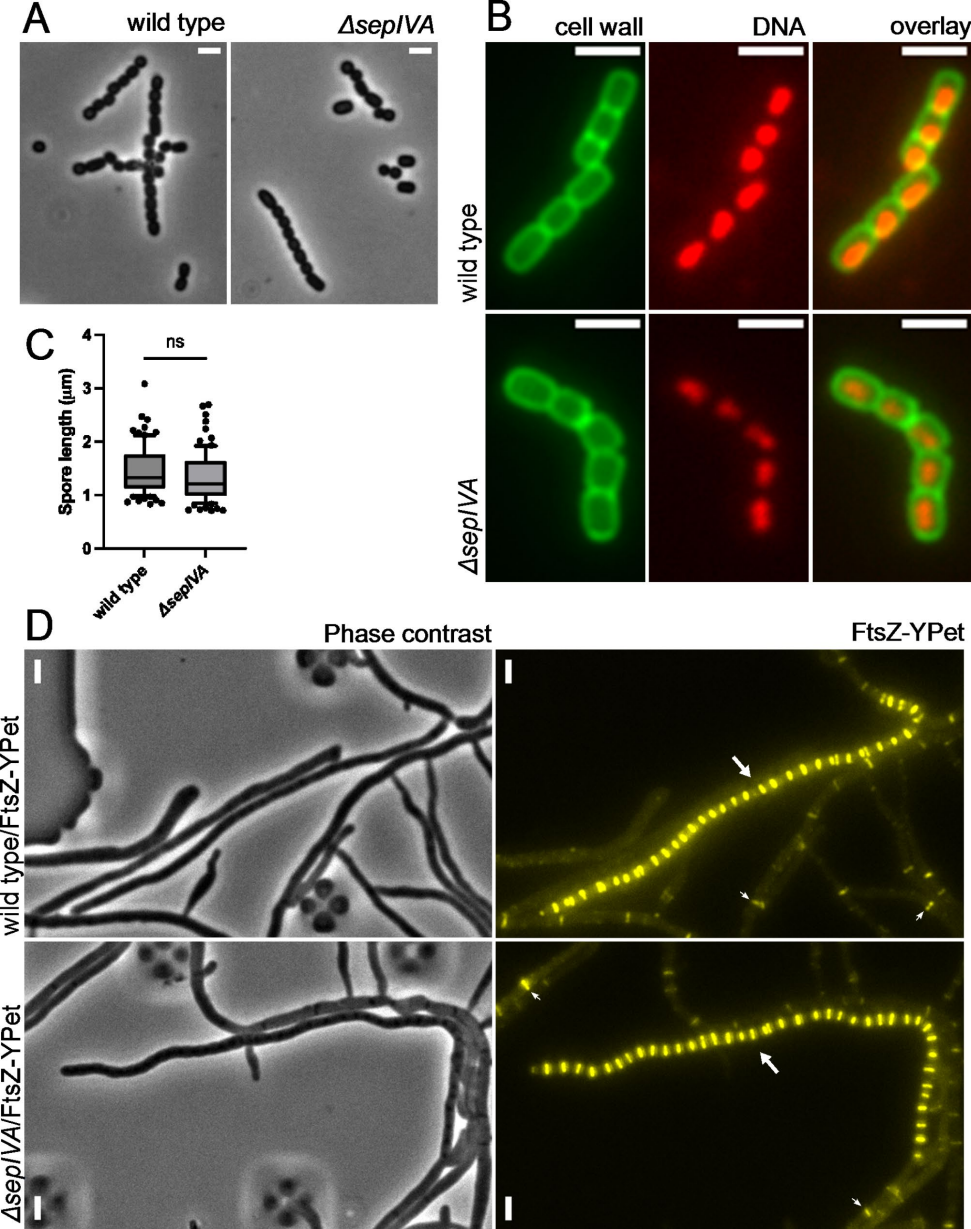
No detectable effect of *sepIVA* on cell division. **A**) Phase contrast micrographs of spore chains of wild type and LUV080 (*ΔsepIVA*) from the surface of colonies after 4–5 days of growth on MYM agar. Scale bars, 2 μm. **B**) Representative micrographs of sporulating aerial hyphal fragments of wild-type strain and LUV080 (*ΔsepIVA*) grown in liquid MYM and stained with cell wall stain WGA-Oregon green (in green) and DNA stain 7-AAD (in red). Scale bars, 2 μm. **C**) Distributions of spore lengths, as determined in micrographs from WGA staining for wild type and LUV080 (*ΔsepIVA*) strains. Eighty-four spores were measured from randomly selected micrographs for each strain. The graph shows median, quartiles and 10–90 percentiles. Statistical significance was calculated using two-tailed unpaired t-test. ns denotes no significance (*P* > 0.05). **D**) Formation of FtsZ rings in sporulating hyphae visualized with an FtsZ-YPet fluorescent protein fusion (yellow). Cells were cultivated in MYM using a microfluidic cell perfusion system. Representative examples of sporulating hyphae with multiple regularly spaced FtsZ rings indicated by large arrow in strains LUV052 (*attBΦBT1*::pKF543[*ftsZ-ypet*]) and LUV125 (*ΔsepIVA attBΦBT1*::pKF543[*ftsZ-ypet*]). Some representative examples of single FtsZ rings in hyphae of vegetative type are indicated by small arrows. Scale bars, 2 μm

To monitor in more detail whether the deletion of *sepIVA* affected sporulation-specific cell division, cultures were allowed to sporulate on MYM agar. Phase-contrast microscopy of material sampled from the aerial mycelium on colony surfaces revealed spores and spore chains of the mutant that were of similar shapes and sizes as observed for the wild-type strain (Fig. 2A). In *Streptomyces.* spp., sporulation-specific cell division, chromosome segregation and cell wall synthesis are tightly coordinated [73]. To observe if deletion of *sepIVA* leads to a defect in any of these processes, we stained DNA (with 7-AAD) and cell wall (with WGA-Oregon green) in sporulating hypha of the mutant. The staining revealed normally condensed and segregated nucleoids in the spores and no clear defects in spore wall appearance or spore shape of the null mutant compared to the wild type (Fig. 2B). Further, no significant difference in the distributions of spore length was found between these strains (Fig. 2C).

To investigate whether deletion of *sepIVA* has any effect on Z-ring assembly, we studied the dynamics of FtsZ in the *sepIVA* mutant strain producing FtsZ-YPet (LUV125) using microfluidics and time-lapse fluorescence microscopy. Both the wild-type parent and the mutant strain, while growing vegetatively, showed dynamic FtsZ rings that visibly moved along hyphae before increasing in intensity and becoming fixed in position. During sporulation, formation of the characteristic arrangements of FtsZ rings in a ladder-like fashion was similarly observed in sporogenic hyphae in both strains (Fig. 2D, Supplementary Movies 1 and 2). Altogether, the results suggest that SepIVA does not have any impact on or role in cell division or FtsZ-ring assembly in *S. venezuelae*.

### SepIVA localizes to tips of growing vegetative hyphae and not to cell division sites

**Fig. 3:**
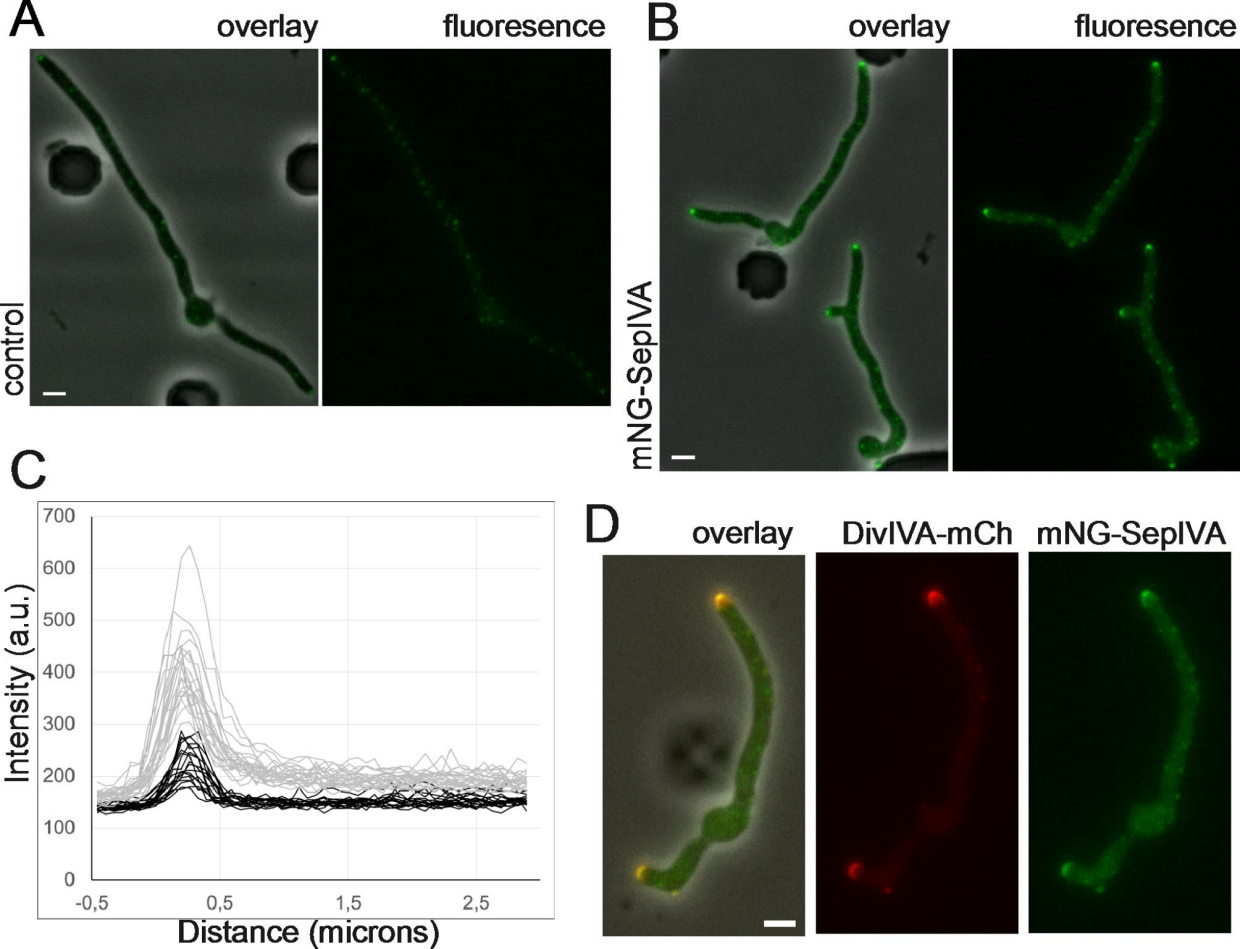
Subcellular localisation of SepIVA. **A**, **B**) Representative phase contrast and fluorescence micrographs of *S. venezuelae* strains carrying empty vector pSS76 **A**) or producing an mNeongreen-SepIVA fusion protein from pKF704. **B**). *S. venezuelae* wild-type strains carrying either pKF704 (with *mNeongreen-sepIVA*) or empty vector pSS76 were cultivated and imaged in a microfluidic cell perfusion system in MYM medium. **C**) Fluorescence intensity profiles along growing hyphae of strains producing mNeongreen or carrying empty vector (example images shown in panels A and B). Profiles were aligned at hyphal apex using the phase contrast intensity profiles. Further analysis of the data is shown in Supplementary Fig. S5. **D**) Representative fluorescence micrographs (and fluorescence images overlaid on phase contrast images) of young hyphae growing out of spores in a microfluidic cell perfusion system in MYM medium. *S. venezuelae* strain LUV189 encodes both mNeongreen-SepIVA and DivIVA-mCherry fusion proteins from plasmid pKF733. Scale bars, 2 μm

We investigated the sub-cellular localization of SepIVA to see if this could give an indication about possible roles in *S. venezuelae*. A strain that produces fluorescent protein mNeonGreen (mNG) fused to the N-terminus of SepIVA was constructed. Expression of the gene fusion was driven by the heterologous and constituive *kasO*p* promoter [62]. By growing a strain expressing this mNG-SepIVA fusion (LUV171) in liquid culture and examining by fluorescence microscopy, we saw a clear localization of SepIVA to the tips of vegetative hyphae (Fig. 3B). The fluorescence intensity of the apical foci was significantly higher than faint foci observed from inherent autofluorescence of the hyphae (Fig. 3C, Supplementary Fig. S5). In addition to apical foci, a significant increase in the level of fluorescence along the hyphae in strain LUV171 compared to the control (Fig. 3C, Supplementary Fig. S5) suggested that SepIVA to some extent also showed a diffuse distribution in the cytoplasm. In time-lapse fluorescence microscopy, mNG-SepIVA localization to the hyphal tips was seen throughout vegetative growth (Movie 3). When co-produced in the same strain, there was clear co-localization between mNG-SepIVA and DivIVA-mCherry at the tips of vegetatively growing hyphae (Fig. 3D). It has previously been shown that lateral hyphal branches emerge from small foci of DivIVA [5], and the majority of these small foci are formed by budding off as daughter polarisomes from the apical polarisome as the hyphal tip extends [25]. Also, mNG-SepIVA foci at hyphal tips show similar dynamics and small foci are often seen on the lateral wall at sites where branches subsequently emerge (Supplementary Movie 3). We did not observe localization of mNG-SepIVA to cell division sites in the time-lapse experiments (Fig. 3, Movie 3). Overall, the data suggest that SepIVA co-localises with DivIVA in apical polarisomes.

### SepIVA interacts with DivIVA in a bacterial two-hybrid system

**Fig. 4:**
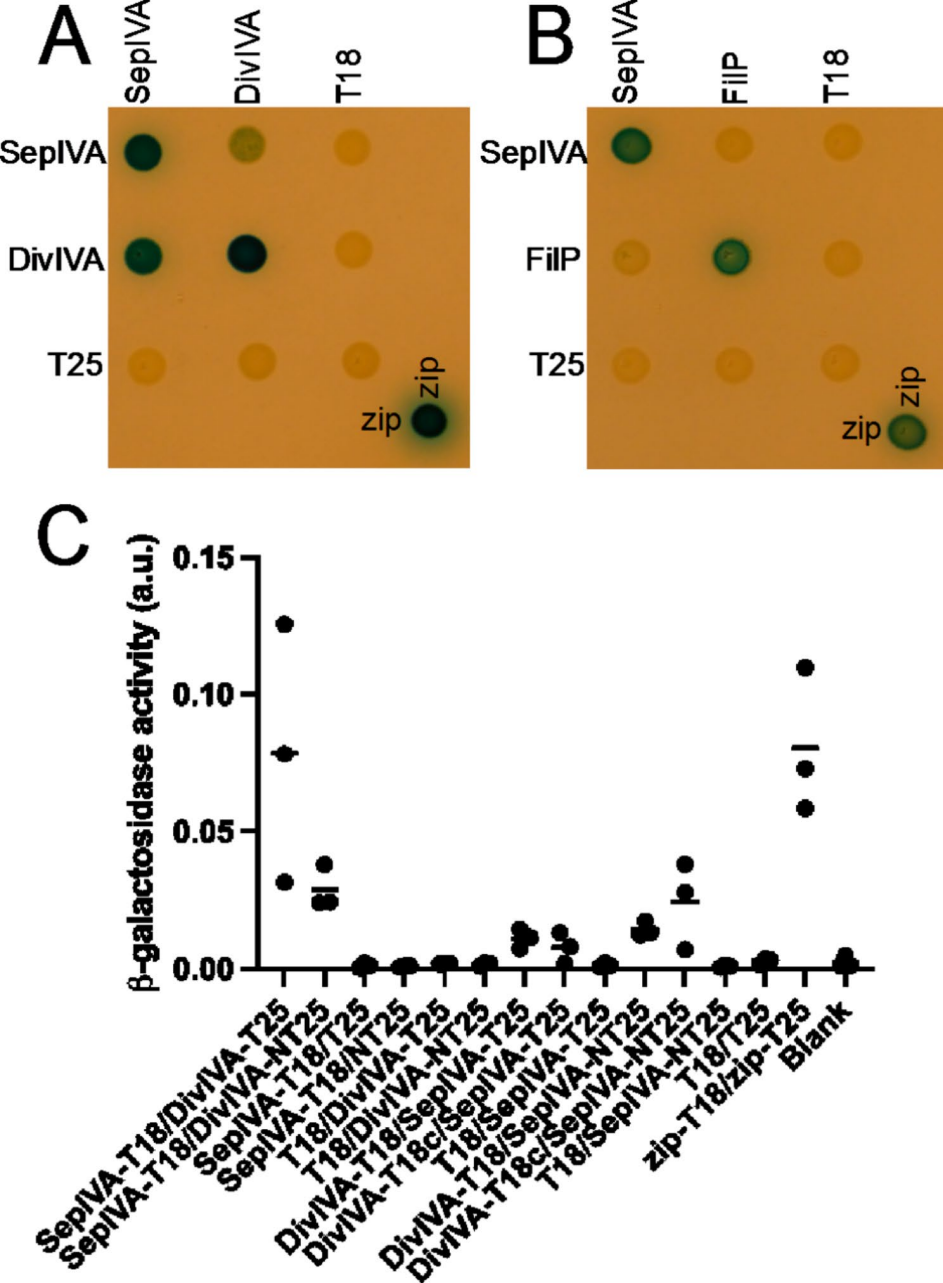
SepIVA interacts with DivIVA. Bacterial two-hybrid assays were performed by growing cultures of E. coli strain BTH101 carrying plasmids producing SepIVA, DivIVA, or FilP fused to the T18 or the T25 domains of adenylate cyclase. **A, B**) Spot-test on LB agar supplemented with X-gal. Overnight cultures of transformants of *E. coli* strain BTH101 carrying pairs of plasmids, based on vectors pUT18 (pUT18c for DivIVA) and pKT25. Representative examples are shown form at least three biological replicates. **C**) β-galactosidase assays from overnight liquid cultures of *E. coli* strain BTH101 transformed with relevant pairs of plasmids (as indicated), testing for interaction between SepIVA and DivIVA. A representative experiment is shown, including mean values of three biological replicates

The sub-cellular localization of SepIVA suggests that it may interact with polarisome proteins. A bacterial two-hybrid system (BACTH) was used to test for interactions with DivIVA and FilP. The latter is a coiled-coil protein implicated in polar growth and cell shape in streptomycetes that carries a DivIVA-like domain and interacts with DivIVA [28, 30]. These results confirm that SepIVA interacts with itself (Fig. 4), as indicated by the Alphafold modelling (Supplementary Fig. S2C) and as expected since both DivIVA and GpsB show homo-oligomerization [11]. Further, SepIVA interacts with DivIVA but not with FilP (Fig. 4). This interaction is consistent with the observed co-localisation between DivIVA and SepIVA at hyphal tips.

### *sepIVA* deletion caused no detectable phenotype

**Fig. 5:**
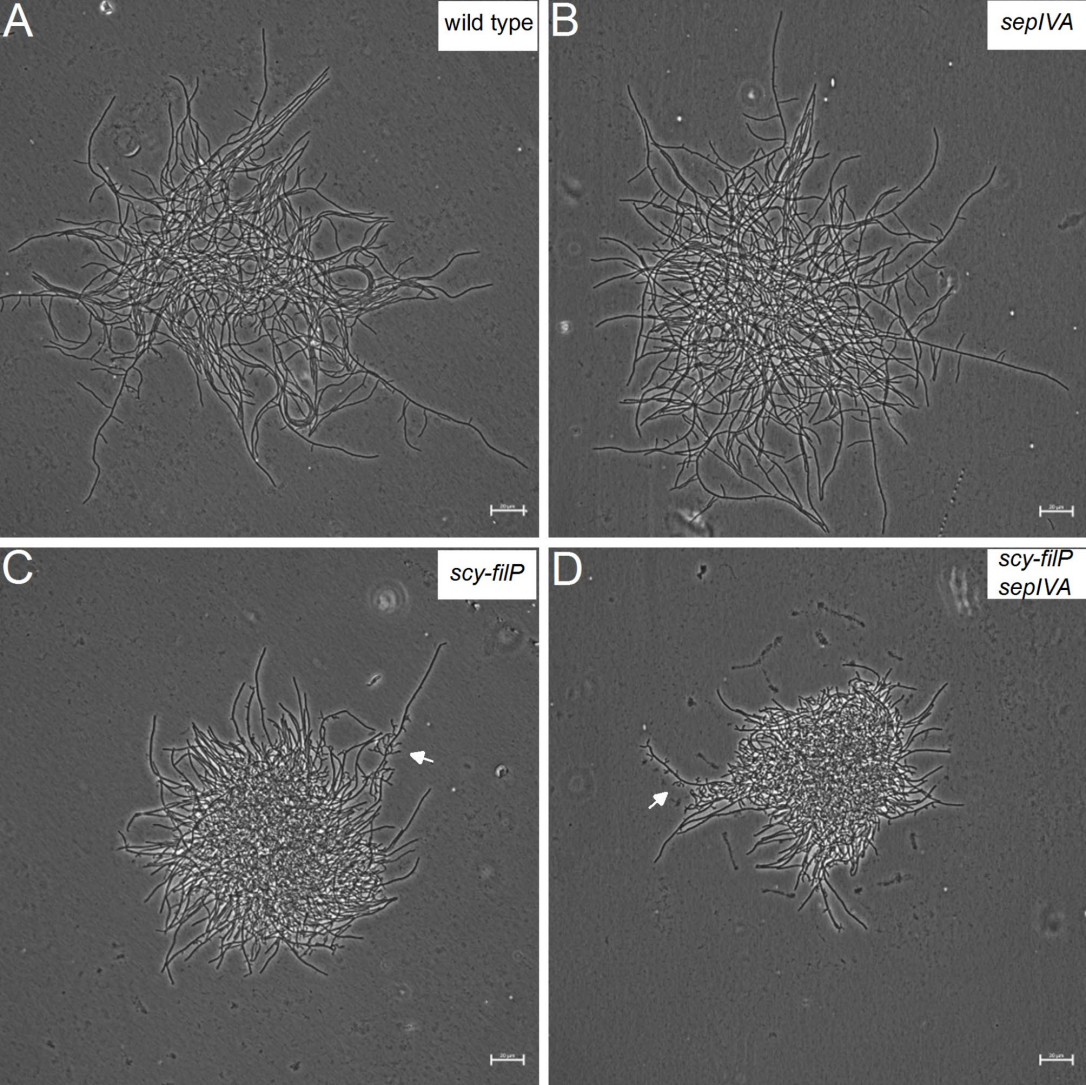
No effect of *sepIVA* on mycelial growth and shape in wild type and *Δ(scy-filP)* mutant backgrounds. Microcolonies of selected strains were allowed to develop on cellophane sheets on MYM agar medium for 16 h at 30 °C. Cellophane sheets were then mounted on agarose-coated slides and observed by phase-contrast microscopy. Strains were **A**) *S. venezuelae* wild-type strain NRRL B-65442; **B**) LUV340 (*ΔsepIVA::apra*); **C**) NA1256 (*Δ(scy-filP)::FRT*); and **D**) LUV339 (*ΔsepIVA::apra Δ(scy-filP)::FRT*). Arrows point to examples of the irregularly shaped and highly branched side branches that are characteristic of *scy* and *scy-filP* mutants. Scale bar, 20 μm

The tip localization of SepIVA suggested that SepIVA is involved in polar growth in *S. venezuelae*. To test whether SepIVA affects hyphal growth pattern and hyphal shape during vegetative stage, a null mutant (LUV080) was grown in MYM medium in a microfluidic cell perfusion system and followed by time lapse microscopy. Microscopic observation of the mutant revealed no obvious effects on hyphal growth pattern, tip extension rates, hyphal shape, or hyphal branching during vegetative growth when compared to the wild-type parent strain (Supplementary Fig. S6). Further, the morphology of *sepIVA* mutant mycelium that developed on cellophane surfaces on MYM or MOPS glucose minimal medium did not differ detectably from the wild-type parent (Fig. 5A, B; Supplementary Fig. S7A, B). Overall, these results show that even though SepIVA has a DivIVA-like domain and localizes in a similar manner as DivIVA, deletion of *sepIVA* does not detectably affect polar growth or determination of cell shape in *S. venezuelae*.

It was further tested whether *sepIVA* may affect the switch to the alternative mode of growth which is referred to as exploratory growth. Under these conditions, hyphae change their mode of growth and colonies extend significantly faster over a surface than under normal growth conditions [53]. However, we saw no difference between wild type and mutant in ability to initiate exploratory growth on YP medium (Supplementary Fig. S8).

Finally, we have investigated whether *sepIVA* affects the peptidoglycan cell wall. SepIVA has a domain architecture that is reminiscent of DivIVA and GpsB. GpsB is an adaptor protein that organizes peptidoglycan synthases and other cell wall-related proteins in Firmicutes [11, 74, 75]. Actinomycete DivIVA has key roles in directing polar cell wall assembly and has been reported to interact with peptidoglycan synthases in mycobacteria and corynebacteria [5, 6, 19, 24]. Against this background, we wondered whether *sepIVA* deletion could affect structure or composition of the peptidoglycan cell wall. Samples were taken of vegetative mycelium in exponential phase of growth of wild type, *sepIVA* mutant, and complemented mutant. The cell wall sacculi were isolated, and their muropeptide profiles were analyzed (Supplementary Fig. S9, Table S4). The results indicated an overall peptidoglycan composition that is in agreement with a previous report on *Streptomyces coelicolor* [76], with a substantial fraction of peptide cross-links being L, D-crosslinks, presence of amidation of D-iGlu to D-iGln, and loss of GlcNAc-MurNAc from some muropeptide dimers, which could indicate peptidoglycan turnover (Table S5). Importantly, however, this investigation failed to detect any significant difference (in unpaired t-tests) between *sepIVA*, its parent strain or the complemented mutant (Supplementary Fig. S9; Tables S4 and S5). Further, the appearance and thickness of the cell wall did not significantly differ between the investigated strains in thin sections of hyphae monitored by transmission electron microscopy (Supplementary Fig. S10). Thus, we failed to detect any cell wall-related phenotype of the *sepIVA* mutants.

### Test for redundancy with other coiled-coil proteins affecting polar growth

In addition to DivIVA, the DivIVA-like coiled-coil proteins FilP and Scy are involved in polar growth in streptomycetes [27–29]. While *divIVA* is essential for viability, deletions of either *scy* or *filP* lead to effects on hyphal growth and morphology, with *scy* giving the stronger effect and appearing to be essentially epistatic on *filP* [27]. Since *sepIVA* encodes another tip-localized DivIVA-like protein, it was possible that *sepIVA* could share partially redundant functions with *scy* and/or *filP*. We tested this hypothesis by investigating whether the inactivation of *sepIVA* would lead to some kind of synthetic phenotype if combined with a *scy-filP* deletion. While the *scy-filP* mutant differed in colony appearance from the wild type on all three tested media as expected, no additional effect of *sepIVA* deletion could be observed in the *scy-filP* mutant background (Supplementary Fig. S4). Mycelial growth at the microscopic level was investigated in microcolonies grown on cellophane membranes placed on surfaces on rich complex MYM agar or MOPS glucose minimal agar. The morphology of hyphae and the resulting mycelium in the wild type is medium-dependent, and hyphae are for example straighter on MYM and more meandering and better dispersed from each other on the minimal medium (Fig. 5 and Supplementary Fig. S7). The *sepIVA* mutant did not differ detectably from the wild-type parent on either of the media. The *scy-filP* deletion strain showed a typical morphology consistent with the previous report [27], where many hyphal branches are poorly growing, irregularly shaped and highly branched, while other hyphae are able to develop and grow relatively normally, leading to microcolonies with a dense core of highly branched hyphae and apparently normal hyphae growing out of the dense core. Many of the side branches on these hyphae show the irregular and highly branched morphology typical of *scy* mutants (arrows in Fig. 5CD). We did not detect any clear difference between the triple *sepIVA scy filP* mutant and the *scy filP* double mutant (Fig. 5 and Supplementary Fig. S6). These experiments did not reveal any synthetic phenotypes, and therefore did not suggest any overlapping roles between *scy-filP* and *sepIVA*.

## Discussion

The subcellular localisation and the interaction of *Streptomyces* SepIVA with DivIVA suggest an association with hyphal tips and possibly an involvement in processes connected to polar growth, even though no clear mutant phenotype could be detected. The findings stand in contrast to the situation in mycobacteria, where SepIVA has been reported to be a cell division protein that is essential for viability [31, 32]. However, it should be noted that Wu et al.. also showed localization of SepIVA to the IMD domains that are associated with assembly of the cell envelope at the cell poles, and it was speculated that the mycobacterial SepIVA may have a regulatory role in cell wall synthesis [32]. The observed differences in the effects of *sepIVA* mutations in rod-shaped mycobacteria compared to hyphal streptomycetes may be related to the difference between these organisms in how polar growth is initiated and how it is related to cell division. In rod-shaped actinomycetes, polar growth is established at the cell poles that are generated by cell division, meaning that there is likely a connection between the cell division machinery and cell elongation. Thus, a protein remaining at the cell pole after division can also affect polar growth. In contrast, polar growth in streptomycetes is independent of cell division [41, 42], and new cell poles are generated *de novo* by lateral branching [25].

Specifically in streptomycetes, four related coiled-coil proteins (DivIVA, Scy, FilP, and SepIVA) are now known to be associated with hyphal tips where cell wall elongation is taking place. Their exact roles and what type of structures they form at the cell poles still remain elusive. DivIVA is essential for growth and both Scy and FilP are associated with distinctive mutant phenotypes, but we were unable to detect any consequences of knocking out *sepIVA*, even when also *scy* and *filP* are absent.

## Conclusion

Although SepIVA is reported to be essential in mycobacteria and orthologues are widespread among actinomycetes, this study shows that SepIVA in *S. venezuelae* is dispensable for viability, and we found no evidence that it would be involved in cell division in this organism. Instead, SepIVA joins a group of three other DivIVA-like proteins that are associated with polar growth in streptomycetes. Further work will hopefully elucidate the role of SepIVA and its relation to the other DivIVA-like proteins. Comparative studies in other actinomycetes should help clarify the intriguing diversity that has evolved in how the machineries for polar growth and cell division are regulated and coordinated in this group of organisms and how this relates to their diversity in cell morphologies and growth habits.

## Electronic supplementary material

Below is the link to the electronic supplementary material.

Supplementary Material 1

Supplementary Material 2

Supplementary Material 3

Supplementary Material 4

Supplementary Material 5

Supplementary Material 6

## Data Availability

The genome sequence datasets generated and analysed during the current study are the European Nucleotide Archive (ENA) at EMBL-EBI under accession number PRJEB81866 (https://www.ebi.ac.uk/ena/browser/view/PRJEB81866). The data for transcription start site mapping that was analysed for generation of Fig. S3 is available at ArrayExpress (accession number E-MTAB-10690), generated and deposited by Mark Buttner, Govind Candra, and Matthew Bush, John Innes Centre, Norwich, UK. Alphafold Multimer models are provided as supplementary files. Other data used and/or analysed during the current study are available from the corresponding author upon reasonable request.
