# Supplementary material for: A dispensable SepIVA orthologue in Streptomyces venezuelae is associated with polar growth and not cell division
Source: BMC Microbiol. 2024 Nov 18;24:481. doi: 10.1186/s12866-024-03625-6 (PMC11571769; doi:10.1186/s12866-024-03625-6)
Supplement: Supplementary file 6 — Supplementary Material 6 [file 12866_2024_3625_MOESM6_ESM.pdf]

Supplementary material for

A dispensable SepIVA orthologue in *Streptomyces venezuelae* is associated with polar growth and not cell division

Beer Chakra Sen[1], Parminder Singh Mavi[1], Oihane Irazoki[2], Susmita Datta[1], Sebastian Kaiser[1], Felipe Cava[2] and Klas Flärdh[1]*

Supplementary figures

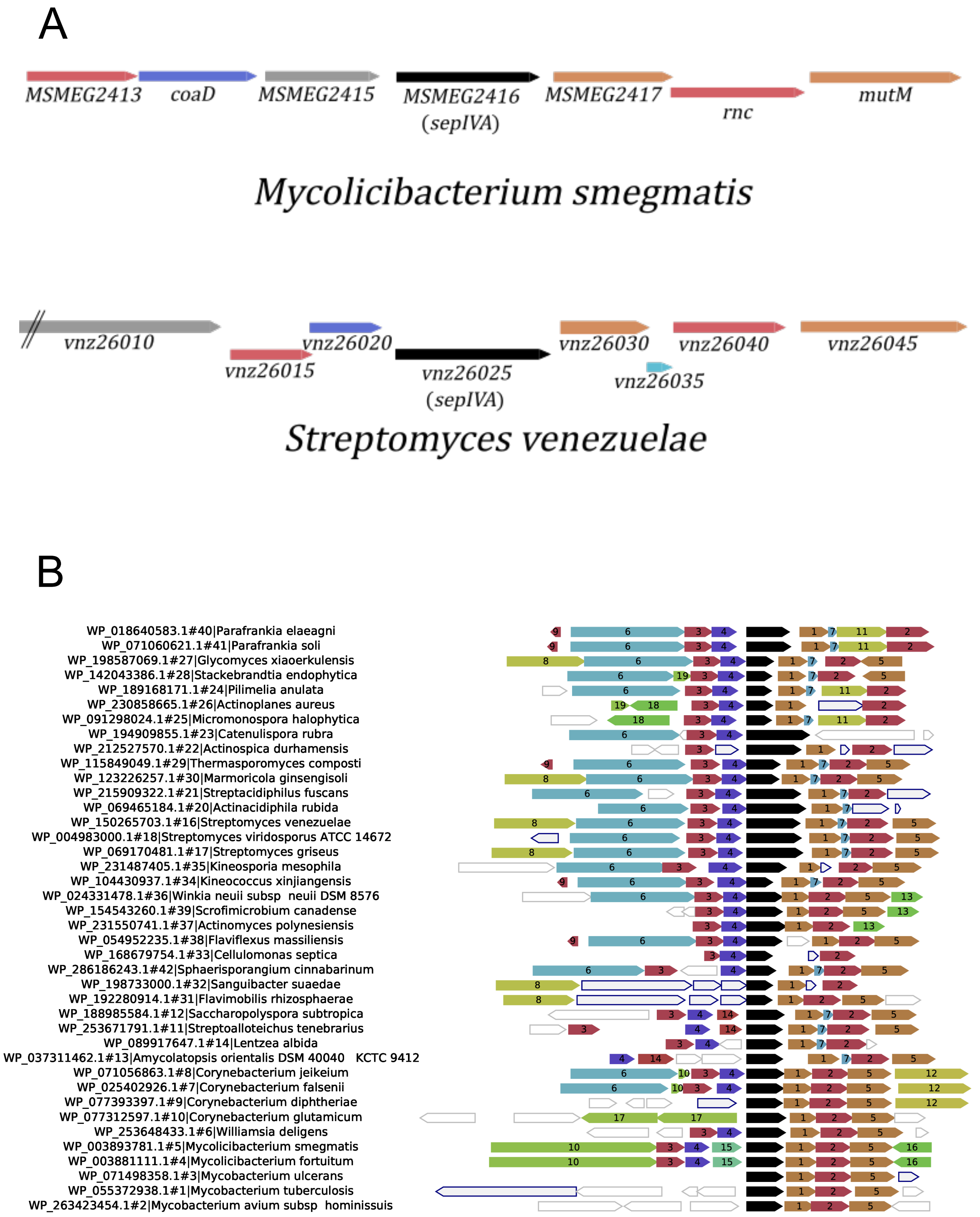

Figure S1. **A)** Schematic view of *sepIVA* loci in *M. smegmatis* and *S. venezuelae* genomes. Flanking genes that show homology between the two species are colour coded in a similar way as in panel B. The *sepIVA* gene is shown in black. **B)** Conservation of gene neighborhoods of the *sepIVA* locus of selected species from all suborders of the Actinomycetales, as detected by the webFlaGs tool (Saha, *et al.*, 2021). Genes encoding SepIVA orthologues are shown in black, and conserved flanking genes are colour coded. Protein encoded by conserved flanking genes are numbered as follows, with annotations based on the *S. venezuelae* NCBI accession number: 1) WP_017947612.1, YceD family protein; 2) WP_015036449.1, Ribonuclease III; 3) WP_019058669.1, 16S rRNA (guanine(966)-N(2))-

methyltransferase RsmD; 4) WP_018102411.1, pantetheine-phosphate adenylyltransferase; 5) WP_015036450.1, bifunctional DNA-formamidopyrimidine glycosylase/DNA-(apurinic or apyrimidinic site) lyase; 6) WP_003997597.1, ATP-dependent DNA helicase RecG, 7) Q9ZBQ8.3, Large ribosomal subunit protein bL32A, 8) WP_015036442.1, DAK2 domain-containing protein. The species and suborders from which the selected 40 putative SepIVA orthologues derive are listed in Supplementary Table S3.

A

```
MSMEG2416       MYRVFEALDELGAIVEEARGVPMTAGCVVPRGDVLELIDDIKDAIPGELDDAQDVLDARD 60
Vnz26025        -VDVQKKLDEIVATVQGARSMPMSASCVVNRAELLALLEEVREALPGSLAQAQELIGGRE 59
                   * : ***: * *: **.:**:*.*** *.::* *::::::*:**.* :**:::..*:

MSMEG2416       SLLREAKEHSESVISGANAEADSVLSHARAEADRLLADAKAQADRMVAEARQHSERMVTE 120
Vnz26025        HLVEQARQEAERIIEAAHAERGSIVSDTQVA-----RQSQEEADRILAEARREAEEVRAE 114
                 *:.:*::.:* :*..*:** .*::*.::.       ::: :***::****:.:*.: :*

MSMEG2416       AREEAARLAAAAKREYEASTGRAKAEADRLIENGNISYEKAIQEGI----KEQQRLVSQT 176
Vnz26025        A--------------------------DDYVDSKLANFEVVLNKTIGSVDRGREKLLGRG 148
                *                          *  ::.   .:* .::: *    : :::*:.:

MSMEG2416       EIVATANAEATRLIDAAHAEADRLRGECDIYVDSKLAEFEEFLNGTLRSVGRGRHQLRTT 236
Vnz26025        PGLDQ-DGYADEDAPEYSADPQTLIQRADEYVDAKLGAFEAVLSKTLEAVGRGRQKLHGR 207
                  :   :. * .      *: : *  ..* ***:**. ** .*. **.:*****::*:

MSMEG2416       AGTHDYVTR--------------------------------------------------- 245
Vnz26025        IATDALGEHMAAQEGLGGPVHTSDSDYLAGLAELADPEPVQIPAQQQAQPQADPYAGYQQ 267
                 .*.    :

MSMEG2416       ------------------------------------------------------------ 245
Vnz26025        VQQQPDPYAGYQQVQQQPDPYAYQQQPQHTDPYGYQQQDPYAYQQPVQQQAHPQQGAALD 327

MSMEG2416       ---------------------- 245
Vnz26025        ETSFFDTSMIDLEQLRRYEQGH 349
```

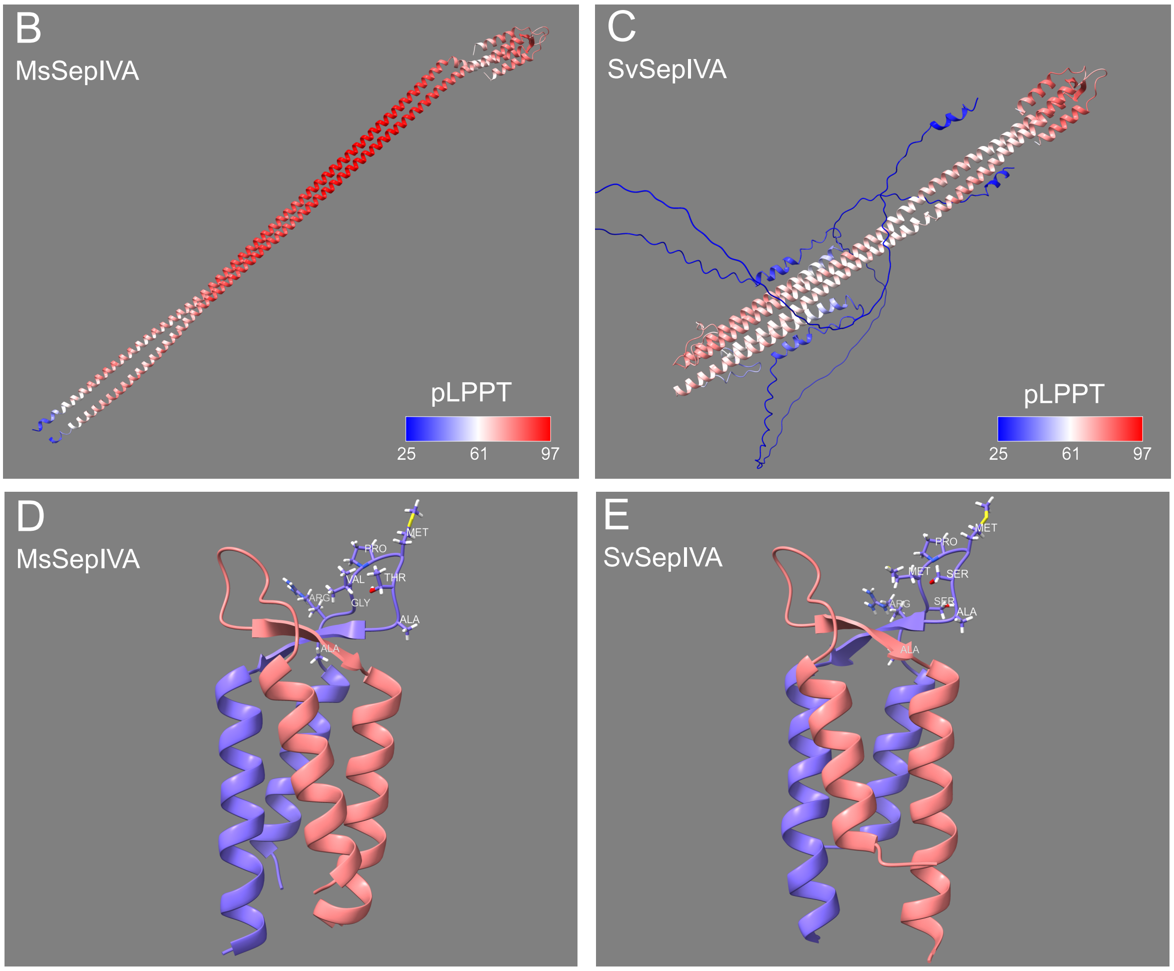

Figure S2. **(A)** Alignment of amino acid sequences of SepIVA orthologues from *M. smegmatis* and *S. venezuelae*, using Clustal Omega. **(B-C)** Models of SepIVA homodimers obtained with Alphafold Multimer. The displayed model of *M. smegmatis* SepIVA (**B**) had iptm+ptm score 0.5 and pDockQ score 0.72, and the *S. venezuelae* SepIVA model (**C**) had iptm+ptm score 0.32 and pDockQ score 0.40. The models are coloured after the pLPPT scores. **(D-E)** View of the conserved N-terminal domain,

annotated as DivIVA-like in the two SepIVA orthologues. Figures generated with ChimeraX (Pettersen, *et al.*, 2021).

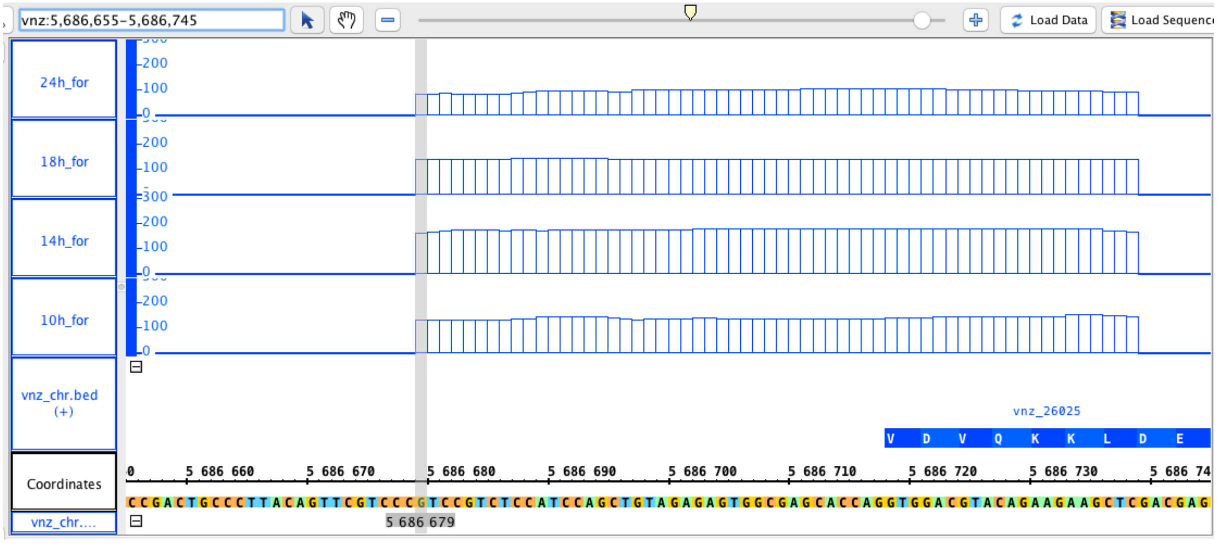

Figure S3. Transcription start site mapping of *sepIVA* in *S. venezuelae*. The figure is based on 5'-triphosphate end-capture RNA-seq data obtained by Matt Bush, John Innes Centre (ArrayExpress, accession number E-MTAB-10690), as described (https://streptomyces.org.uk/vnz_tss.html). Normalised coverage by TSS reads on the forward strand, subtracted by Normalised coverage by control reads on the forward strand is displayed. The grey vertical line at position 5,686,679 indicates the estimated transcription start site. As described, for time points along a liquid culture time course for *S. venezuelae* were analysed: 10 hr, 14 hr, 18 hr and 24 hr to represent the transition throughout development (vegetative, pre-sporulation, onset of sporulation and mid/late sporulation).

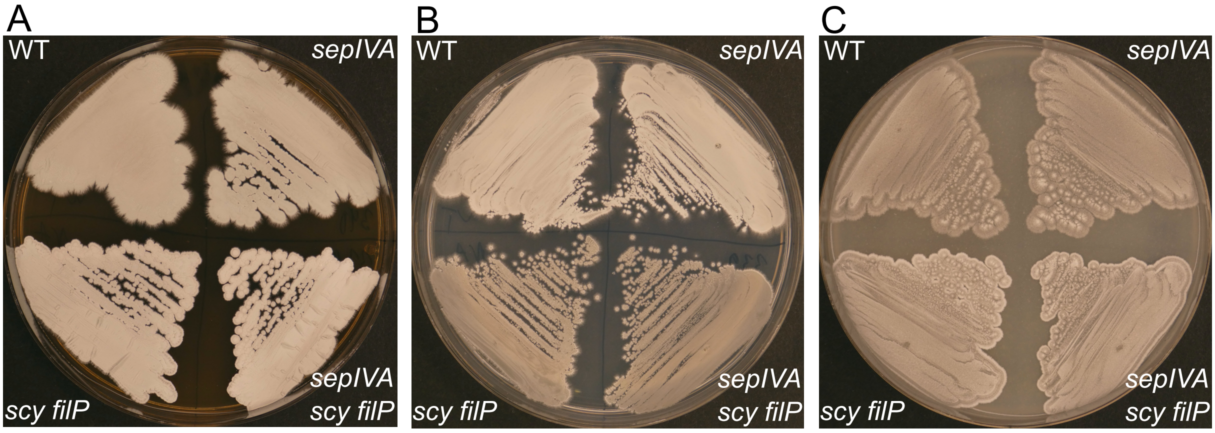

Figure S4. No effect of *sepIVA* on plate phenotypes on different media and in both wild type and *Δ(scy-filP)* mutant background. Spores were streaked on rich MYM agar (**A**), MOPS glucose minimal medium (**B**), and chitin agar medium (**C**). Plates were incubated for 5 days at 30°C before photographs were taken. Strains were *S. venezuelae* wild-type strain NRRL B-65442; LUV340 (*ΔsepIVA::apra*); NA1256 (*Δ(scy-filP)::FRT*); and LUV339 (*ΔsepIVA::apra Δ(scy-filP)::FRT*).

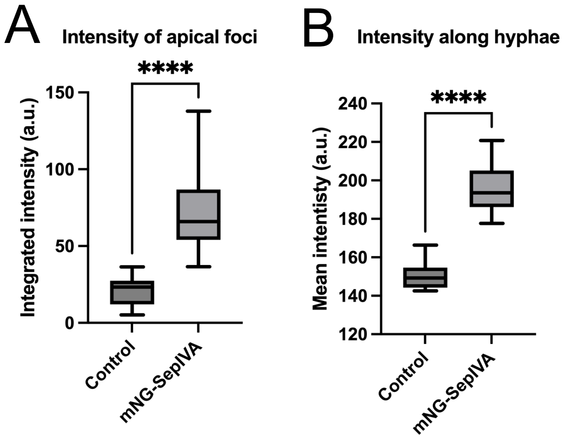

Figure S5. Fluorescence intensity signals from mNG-SepIVA compared to the autofluorescence background seen in the control strains carrying only empty vector. Representative images from the data set and a compilation of the fluorescence profiles along hyphae that were analysed are shown in Figure 4. **A)** The fluorescence intensity profiles were integrated from 0 to 0.5 μm, setting baseline for each profile to the average intensity value for the profile from 1.0 to 2.5 μm. This shows the difference in intensity for the apical foci between the mNG-SepIVA strain and the control. **B)** Difference in intensity along hyphae between strain expressing mNG-SepIVA and the autofluorescence control. Intensity values from the fluorescence profiles were averaged from 1.0 to 2.5 μm from the tip. The graphs show also the results on unpaired t tests with Welch's correction, showing that the differences of means are significant (P<0.0001).

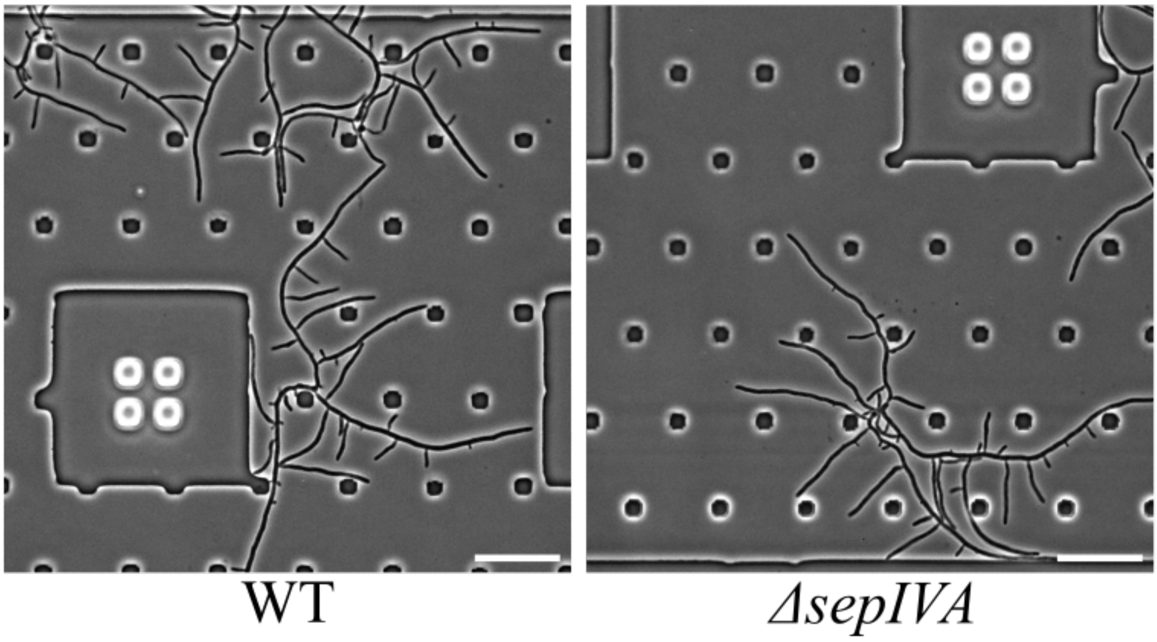

Figure S6. Cultivation of *sepIVA* mutant strain LUV080 and its wild-type parent of *S. venezuelae* in a microfluidic cell perfusions system (CellASIC ONIX2). Representative images of rapidly growing hyphae in MYM medium are shown. No overt differences in hyphal growth or morphology between mutant and wild type were observed at any stage of growth. Size bars, 20 μm.

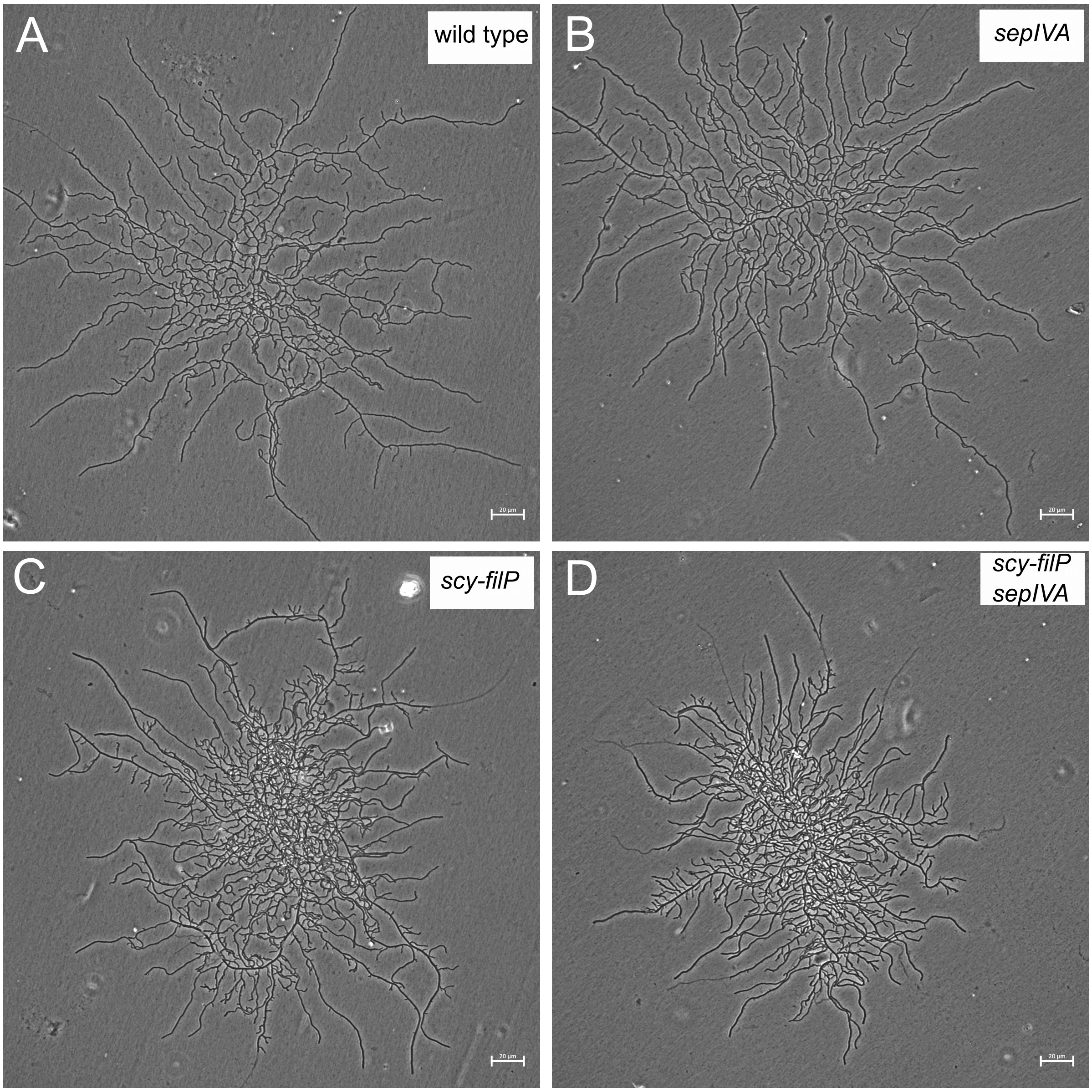

Figure S7. No effect of *sepIVA* on mycelial growth and shape in wild type and *Δ(scy-filP)* mutant backgrounds. Microcolonies of selected strains were allowed to develop on cellophane sheets on MOPS glucose minimal agar medium for 16 h at 30°C. The cellophane sheets were then mounted on agarose-coated slides and observed by phase-contrast microscopy. Strains were **A)** *S. venezuelae* wild-type strain NRRL B-65442; **B)** LUV340 (*ΔsepIVA::apra*); **C)** NA1256 (*Δ(scy-filP)::FRT*); and **D)** LUV339 (*ΔsepIVA::apra Δ(scy-filP)::FRT*). Scale bar, 20 μm.

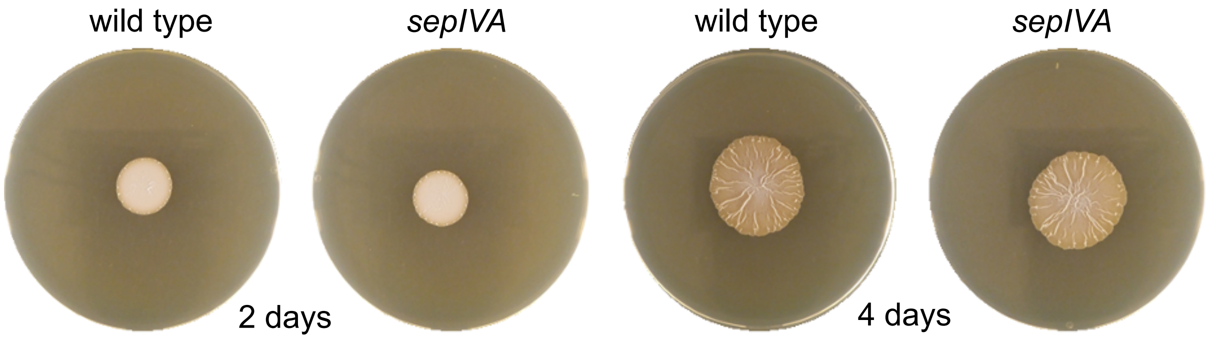

Figure S8. No effect of *sepIVA* on exploratory growth. *S. venezuelae* wild-type strain NRRL B-65442 and its *ΔsepIVA::apra* derivative, strain LUV340, were spotted (5 µl diluted spore suspension) in the middle of 88 mm Petri dishes with YP agar medium and incubated at 30°C over several days to allow exploratory growth to develop and spread over the agar medium. No difference was noted between wild type and the mutants in development of exploratory growth.

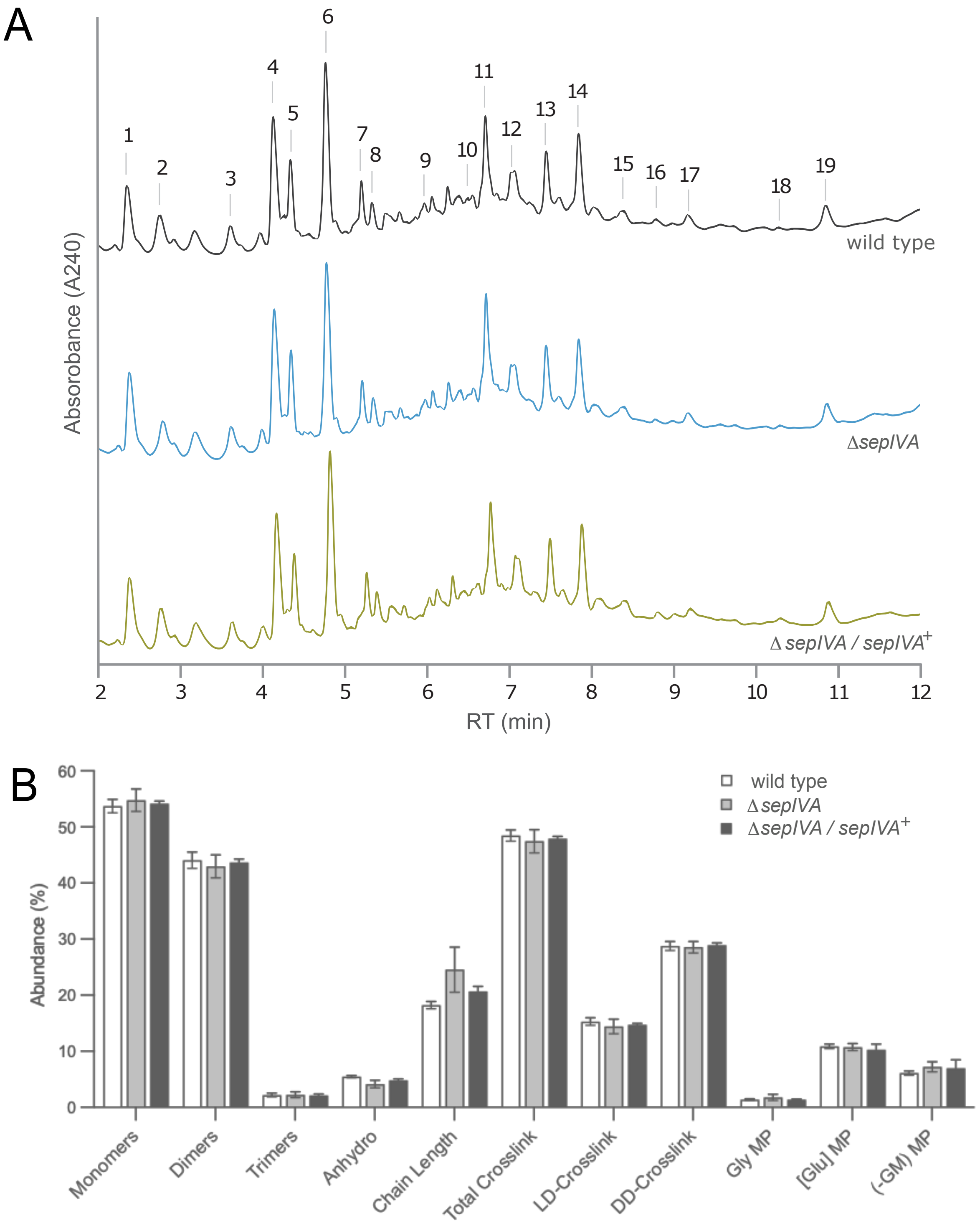

Figure S9. Analysis of peptidoglycan (PG) and muropeptide profiles of *sepIVA* mutant compared to wild type and *in trans* complemented mutant. **A)** PG profiles. Representative UPLC chromatograms of wild-type *S. venezuelae* NRRL B-65442 (black), *sepIVA* mutant strain LUV080 (blue) and complemented mutant strain LUV112 (*ΔsepIVA*::pKF756[*sepIVA*$^+$]; yellow). Identified muropeptides are labelled, with numbering and quantification shown in Table S4. **B)** PG composition of the analysed strains is summarised. See also Table S5.

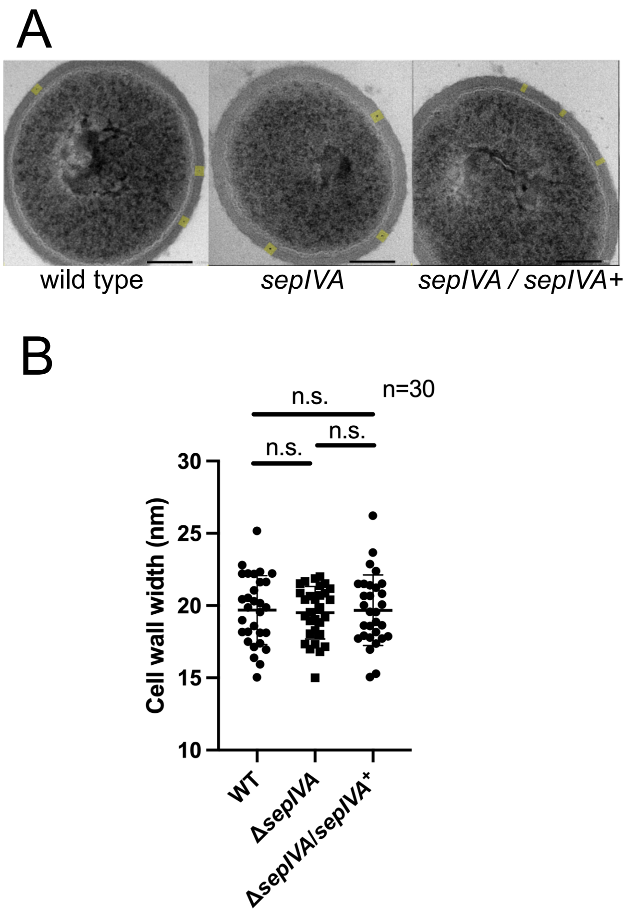

Figure S10. No effect of *sepIVA* on cell wall thickness in vegetative hyphae. Vegetive mycelium from growing liquid cultures in MYM medium, OD_{600} at 1.0, was prepared for thin sectioning and transmission electron microscopy. Images that appear to be cross-sections of vegetative hyphae, perpendicular to the length axis, were selected, and the thickness of the peptidoglycan cell wall layer was measured, with help of an intensity plot along a line drawn across the cell envelope, as indicated in representative images (**A**). Data are plotted with mean and standard deviations (**B**), and two-tailed unpaired t-tests revealed no significant (n.s.) differences between the mean values ($P>0.05$). The strains were wild-type *S. venezuelae* NRRL B-65442, *sepIVA* mutant strain LUV080, and the mutant complemented by *sepIVA* expressed from *kasOp** from plasmid pKF756.

Supplementary tables

Table S1: Plasmids used in this study

Vector/ construct	Description[1]	Reference or source
pIJ773	Source for apramycin resistance cassette, *aac(3)IV-oriT*	(Gust*, et al.*, 2003)
pIJ10770	Derivative of pMS82; Hyg^r	(Schlimpert*, et al.*, 2017)
pKF537	Synthesized version of mNeonGreen gene with codon choice optimized for *Streptomyces*; Amp^r	K. Flärdh
pKF543	Derivative of pIJ10770 encoding *S. venezuelae* FtsZ-Ypet fusion; Hyg^r	(Sen*, et al.*, 2019)
pKF601	*divIVA* in pUT18C; Amp^r	This work
pKF603	*divIVA* in pKT25; Kan^r	This work
pKF651	Derivative of cosmid pKF709 with *sepIVA* replaced with apramycin resistance cassette, *ΔsepIVA*::*apra*; Apr^r, Kan^r	This work
pKF652	Derivative of pIJ10770 containing *sepIVA*, complementation plasmid; Hyg^r	This work
pKF699	Derivative of pSS76 with *kasOp** driving expression of an *mNeongreen-cvnD2* fusion. Source of *mNeongreen* is pKF537; Hyg^r	K. Flärdh
pKF703	Derivative of pSS76 containing *kasOp*-mNeongreen-sepIVA*; Hyg^r	This work
pKF705	*sepIVA* in pUT18; Amp^r	This work
pKF707	*sepIVA* in pKT25; Kan^r	This work
pKF708	*sepIVA* in pKNT25; Kan^r	This work
pKF709	Cosmid 3-B07 containing *sepIVA*; Amp^r, Kan^r	M.J. Buttner, John Innes Centre, Norwich, U.K.
pKF733	Derivative of pSS204, carrying both P_{kasO}-*mNeongreen-sepIVA* and *divIVA-mCherry*, Hyg^r	This work
pKF756	*sepIVA* under control of *kasOp** in derivative of pSS76; Hyg^r	This work
pKNT25	Plasmid for bacterial two hybrid assay, C-terminal *cyaAT25* fusion; Kan^r	(Karimova*, et al.*, 1998)
pKT25	Plasmid for bacterial two hybrid assay, N-terminal *cyaAT25* fusion; Kan^r	(Karimova*, et al.*, 1998)
pMS82	Vector that integrates at ϕBT1 *attB* site, Hyg^r	(Gregory*, et al.*, 2003)
pNA1286	*filP* in pKNT25; Kan^r	(Fröjd & Flärdh, 2019)
pNA1290	*filP* in pUT18C; Amp^r	(Fröjd & Flärdh, 2019)
pSS76	Derivative of pMS82 with *kasOp** promoter; Hyg^r	S. Schlimpert, John Innes Centre, Norwich, U.K.
pSS204	pIJ10770 containing *divIVA-mCherry* driven by the *divIVA* promoter; Hyg^r	S. Schlimpert, John Innes Centre, Norwich, U.K.
pUT18	Plasmid for bacterial two hybrid assay; C-terminal *cyaAT18* fusion; Amp^r	(Karimova*, et al.*, 1998)
pUT18C	Plasmid for bacterial two hybrid assay; N-terminal *cyaAT18* fusion; Amp^r	(Karimova*, et al.*, 1998)

[1]Amp^r, ampicillin resistance; Apr^r, apramycin resistance; Hyg^r, Hygromycin resistance; Kan^r, Kanamycin resistance

Table S2: Primers used in this study

Primers	Sequence (5'→3')	Comments
KF435	GGGCAGGATAGGTGAAGTAGG	PCR verification of insertion of apramycin resistance cassette and *sepIVA* deletion
KF536	GGATGTGCTGCAAGGCGATT	Sequencing inserts in pKT25
KF538	CTTGATGCCATCGAGTACG	For sequencing inserts in pKTN25
KF541	GTGTGGAATTGTGAGCGGAT	Sequencing inserts in pUT18 and pKNT25
KF542	TTCCACAACAAGTCGATGCG	Sequencing inserts in pUT18
KF1246	CCTCTGACCCCTGACCCCGTC	Sequencing inserts in pIJ10770
KF1272	GAGTTGGTAGCTCTTGATCCGGC	Sequencing inserts in pIJ10770
KF1370	AATATTTCTAGAGCCGCTGACCCCCGAGGAC	Amplification of *divIVA* to clone in pUT18C or pKT25, with XbaI site
KF1371	TATATTGGTACCCAGTTGTCGTCCTCGTCGA	Amplification of *divIVA* to clone in pUT18C or pKT25, with KpnI site
KF1568	CTGGGCGAAGCGTCAAGTATCCTGGCTCTTCGGTCGCGCTATGCTACATCTCTCGAGTTAATTA	Primer for λ Red recombineering of *sepIVA*
KF1569	TGCAGCTCGTAGTCGAAGTCGCTGACGGCGCGCAGTCCCTTGACGCGATGATCATATGAGAGAATCTAAGGTACC	Primer for λ Red recombineering of *sepIVA*
KF1570	CAACGGCCTCACGGGCGTG	PCR verification of *sepIVA* deletion
KF1571	CCGACTCAAGACGGAGATCGAT	PCR verification of *sepIVA* deletion
KF1574	CCCGGGCATATGTACACGTCGCGGTGATGATCAA	Construction of complementation vector with NdeI site
KF1575	CCCGGGGTACCACGCTTCGCCCAGGGCCCA	Construction of complementation vector with KpnI site
KF1579	AGCGCGCCCTCGAGCTTCGCCCAGGGCCCAATCG	Primer for pKF748 cloning
KF1646	TTACCCGGGCTTAAGGTGGACGTACAGAAGAAGC	Amplification of *sepIVA* to construct N-terminal fusion with mNeonGreen, with AflII site
KF1647	ATTACTATAAGCTTTCAGTGTCCCTGTTCGTACCG	Amplification of *sepIVA* to construct N-terminal fusion with mNeonGreen, with HindIII site
KF1656	CCCGGATTCTAGAGGTGGACGTACAGAAGAAGCTCG	Amplification of *sepIVA* to clone into pUT18 or pKT25 or pKNT25, with XbaI site
KF1657	CCCGGGGGTACCGGGTGTCCCTGTTCGTACCGGCGC	Amplification of *sepIVA* to clone into pUT18 or pKT25 or pKNT25, with KpnI site
KF1727	ATGGTTACCTCGCCTCTGAC	Primer for pKF748 cloning
KF1752	TCAATATCATGATCTTTATAATCGCCATCATGATCTTTATAATCATGCATCTTAAGCATATGGTCACCTCTTCAACTC	Primer for pKF748 cloning
KF1776	AGGTGACCATATGGTGGACGTACAGAAGAAGCTCG	Primer for pKF757 cloning
KF1777	AATTAATCAATGCATGTGTCCCTGTTCGTACCGGC	Primer for pKF757 cloning

[1]The underlined regions denote different restriction sites.

Table S3: Accession numbers, species and Actinomycetota suborders of the SepIVA orthologues used in webFlaGs analysis

Suborder	Species	NCBI accession of SepIVA orthologue
Actinomycineae	*Winkia neuii*	WP_024331478.1
Actinomycineae	*Flaviflexus massiliensis*	WP_054952235.1
Actinomycineae	*Scrofimicrobium canadense*	WP_154543260.1
Actinomycineae	*Actinomyces polynesiensis*	WP_231550741.1
Catenulisporinae	*Catenulispora rubra*	WP_194909855.1
Catenulisporinae	*Actinospica durhamensis*	WP_212527570.1
Corynebacterineae	*Mycolicibacterium fortuitum*	WP_003881111.1
Corynebacterineae	*Mycolicibacterium smegmatis*	WP_003893781.1
Corynebacterineae	*Corynebacterium falsenii*	WP_025402926.1
Corynebacterineae	*Mycobacterium tuberculosis*	WP_055372938.1
Corynebacterineae	*Corynebacterium jeikeium*	WP_071056863.1
Corynebacterineae	*Mycobacterium ulcerans*	WP_071498358.1
Corynebacterineae	*Corynebacterium glutamicum*	WP_077312597.1
Corynebacterineae	*Corynebacterium diphtheriae*	WP_077393397.1
Corynebacterineae	*Williamsia deligens*	WP_253648433.1
Corynebacterineae	*Mycobacterium avium*	WP_263423454.1
Frankineae	*Parafrankia elaeagni*	WP_018640583.1
Frankineae	*Parafrankia soli*	WP_071060621.1
Glycomycineae	*Stackebrandtia endophytica*	WP_142043386.1
Glycomycineae	*Glycomyces xiaoerkulensis*	WP_198587069.1
Kineosporiineae	*Kineococcus xinjiangensis*	WP_104430937.1
Kineosporiineae	*Kineosporia mesophila*	WP_231487405.1
Micrococcineae	*Cellulomonas septica*	WP_168679754.1
Micrococcineae	*Flavimobilis rhizosphaerae*	WP_192280914.1
Micrococcineae	*Sanguibacter suaedae*	WP_198733000.1
Micromonosporineae	*Micromonospora halophytica*	WP_091298024.1
Micromonosporineae	*Pilimelia anulata*	WP_189168171.1
Micromonosporineae	*Actinoplanes aureus*	WP_230858665.1
Propionibacterineae	*Thermasporomyces composti*	WP_115849049.1
Propionibacterineae	*Marmoricola ginsengisoli*	WP_123226257.1
Pseudonocardineae	*Amycolatopsis orientalis*	WP_037311462.1
Pseudonocardineae	*Lentzea albida*	WP_089917647.1
Pseudonocardineae	*Saccharopolyspora subtropica*	WP_188985584.1
Pseudonocardineae	*Streptoalloteichus tenebrarius*	WP_253671791.1
Streptomycineae	*Streptomyces viridosporus*	WP_004983000.1
Streptomycineae	*Streptomyces griseus*	WP_069170481.1
Streptomycineae	Actinacidiphila rubida	WP_069465184.1
Streptomycineae	Streptomyces venezuelae	WP_150265703.1
Streptomycineae	Streptacidiphilus fuscans	WP_215909322.1
Streptosporanginae	Sphaerisporangium cinnabarinum	WP_286186243.1

Table S4. Muropeptide composition

Peak	Muropeptide[1]	Time	wild type	Δ*sepIVA*	Δ*sepIVA* / *sepIVA*⁺
1	Tri	2.45	10.81 (±0.29)	11.63 (±0.40)	10.61(±0.29)
2	Di	2.997	5.26 (±0.26)	6.78 (±1.91)	5.75 (±0.12)
3	TetraTri(3-3)(-GM)	3.836	3.15 (±0.06)	3.40 (±0.22)	3.22 (±0.23)
4	Tetra	4.319	14.15 (±1.01)	15.07 (±0.37)	13.82 (±0.29)
5	TetraTetra[Glu]	4.496	5.64 (±0.15)	6.11 (±0.37)	5.23 (±0.06)
6	Penta	4.921	20.21 (±0.48)	18.63 (±0.50)	20.72 (±0.34)
7	TetraTetra(-GM)	5.311	2.96 (±0.34)	3.82 (±0.57)	3.75 (±1.04)
8	Penta[Glu]	5.457	2.60 (±0.26)	2.23 (±0.07)	2.52 (±0.15)
9	TriTri	5.761	1.19 (±0.19)	0.70 (±0.40)	1.25 (±0.04)
10	TetraTetra[Gly4]	6.478	0.77 (±0.08)	0.70 (±0.37)	0.71 (±0.02)
11	TetraTri(3-3)	6.663	6.14 (±0.59)	6.61 (±0.20)	6.19 (±0.14)
12	PentaTetra[Gly5]	6.915	0.62 (±0.04)	0.83 (±0.09)	0.66 (±0.07)
13	TetraTetra	7.569	5.95 (±0.12)	5.98 (±0.26)	6.10 (±0.04)
14	PentaTetra	7.982	10.22 (±0.27)	8.47 (±0.40)	9.99 (±0.23)
15	PentaTetra[Glu]	8.573	2.62 (±0.11)	2.40 (±0.11)	2.51 (±0.80)
16	Penta^{Anh}	9.016	0.68 (±0.15)	0.44 (±0.02)	0.78 (±0.13)
17	Trimer[2]	9.414	1.61 (±0.27)	1.64 (±0.17)	2.00 (±0.33)
18	Trimer[2]	10.576	0.60 (±0.18)	0.61 (±0.27)	0.13 (±0.19)
19	TetraTri(3-3)^{Anh}	11.159	4.81 (±0.05)	3.70 (±0.54)	4.07 (±0.09)

[1]Modifications: (-GM), loss of NAM-NAG; [Glu], presence of Glutamic acid (Glu) instead of Glutamine (Gln); [Gly4] or [Gly5], Gly at position 4 or 5 instead of D-Ala.
[2] Unidentified trimer. Peaks corresponding to trimers were excluded from specific crosslink type analysis but included in the total crosslink quantification.

Table S5. PG composition (relative abundance given as % of total area).

	wild type	Δ*sepIVA*	Δ*sepIVA* / *sepIVA*⁺
**Monomers**	53.73 (±0.99)	54.79 (±1.64)	54.20 (±0.37)
**Dimers**	44.07 (±1.19)	42.97 (±1.68)	43.67 (±0.51)
**Trimers**	2.20 (±0.26)	2.25 (±0.41)	2.13 (±0.22)
**Anhydro**	5.49 (±0.16)	4.14 (±0.56)	4.85 (±0.168)
**Chain Length**	18.22 (±0.52)	24.57 (±3.29)	20.66 (±0.73)
**Total Crosslink**	48.48 (±0.82)	47.46 (±1.70)	47.93 (±0.33)
**LD-Crosslink**	15.30 (±0.55)	14.41 (±1.06)	14.73 (±0.21)
**DD-Crosslink**	28.77 (±0.64)	28.55 (±0.83)	28.94 (±0.33)
**Gly MP**	1.39 (±0.11)	1.78 (±0.45)	1.36 (±0.08)
**[Glu] MP**	10.87 (±0.32)	10.75 (±0.50)	10.25 (±0.84)
**(-GM) MP**	6.11 (±0.31)	7.21 (±0.74)	6.97 (±1.25)

Legends to Supplementary Files and Movies

Supplementary file 1: Movie 1: Time-lapse fluorescence microscopy to follow FtsZ-YPet localization in wild-type *S. venezuelae*. The movie consists of fluorescence images of FtsZ-YPet overlaid on the corresponding phase-contrast images of the strain LUV052 ($attB_{\phi BT1}$::pKF543[*ftsZ-ypet*]). Cells were grown in CellASIC ONIX2 microfluidic system and monitored with fluorescence and phase-contrast microscopy. After an initial period (4-6 hours) of vegetative growth, spent medium was pumped to induce sporulation. Time interval between each frame is 10 min. Scale bar, 5 µm.

Supplementary file 2: Movie 2: Time-lapse fluorescence microscopy to follow FtsZ-YPet localization in *sepIVA* mutant of *S. venezuelae*. The movie consists of fluorescence images of FtsZ-YPet overlaid on the corresponding phase-contrast images of strain LUV125 (*ΔsepIVA* $attB_{\phi BT1}$::pKF543[*ftsZ-ypet*]). Cells were grown in CellASIC ONIX2 microfluidic system and monitored with fluorescence and phase-contrast microscopy. After an initial period (4-6 hours) of vegetative growth, spent medium was pumped to induce sporulation. Time interval between each frame is 10 min. Scale bar, 5 µm.

Supplementary file 3: Movie 3: Time-lapse fluorescence microscopy to follow mNeonGreen-SepIVA localization in *S. venezuelae*. The movie consists of fluorescence images of mNeonGreen-SepIVA overlaid on the corresponding phase-contrast images of the strain LUV169 ($attB_{\phi BT1}$::pKF703[P_{kasO}-*mNeongreen-sepIVA]*). Cells were grown in CellASIC ONIX2 microfluidic system and monitored with fluorescence and phase-contrast microscopy. After an initial period (4-6 hours) of vegetative growth, spent medium was pumped to induce sporulation. Time interval between each frame is 10 min. Scale bar, 5 µm.

Supplementary file 4: Alphafold multimer prediction of a homodimer of *M. smegmatis* SepIVA. Model generated at the LU-fold facility at Lund University (https://www.medicine.lu.se/research-and-research-studies/house-infrastructure/list-research-infrastructures/lu-fold). iptm+ptm score 0.5 and pDockQ score 0.72

Supplementary file 5: Alphafold multimer prediction of a homodimer of *S. venezuelae* SepIVA. Model generated at the LU-fold facility at Lund University (https://www.medicine.lu.se/research-and-research-studies/house-infrastructure/list-research-infrastructures/lu-fold). iptm+ptm score 0.32 and pDockQ score 0.40

References

Fröjd MJ & Flärdh K (2019) Apical assemblies of intermediate filament-like protein FilP are highly dynamic and affect polar growth determinant DivIVA in *Streptomyces venezuelae*. *Molecular Microbiology* **112**: 47-61.

Gregory MA, Till R & Smith MC (2003) Integration site for *Streptomyces* phage phiBT1 and development of site-specific integrating vectors. *Journal of Bacteriology* **185**: 5320-5323.

Gust B, Challis GL, Fowler K, Kieser T & Chater KF (2003) PCR-targeted *Streptomyces* gene replacement identifies a protein domain needed for biosynthesis of the sesquiterpene soil odor geosmin. *Proceedings of the National Academy of Science, USA* **100**: 1541-1546.

Karimova G, Pidoux J, Ullmann A & Ladant D (1998) A bacterial two-hybrid system based on a reconstituted signal transduction pathway. *Proceedings of the National Academy of Science, USA* **95**: 5752-5756.

Pettersen EF, Goddard TD, Huang CC, Meng EC, Couch GS, Croll TI, Morris JH & Ferrin TE (2021) UCSF ChimeraX: Structure visualization for researchers, educators, and developers. *Protein Science* **30**: 70-82.

Saha CK, Sanches Pires R, Brolin H, Delannoy M & Atkinson GC (2021) FlaGs and webFlaGs: discovering novel biology through the analysis of gene neighbourhood conservation. *Bioinformatics* **37**: 1312-1314.

Schlimpert S, Wasserstrom S, Chandra G, Bibb MJ, Findlay KC, Flärdh K & Buttner MJ (2017) Two dynamin-like proteins stabilize FtsZ rings during *Streptomyces* sporulation. *Proceedings of the National Academy of Science, USA* **114**: E6176-E6183.

Sen BC, Wasserstrom S, Findlay KC, Söderholm N, Sandblad L, von Wachenfeldt C & Flärdh K (2019) Specific amino acid substitutions in β strand S2 of FtsZ cause spiraling septation and impair assembly cooperativity in *Streptomyces*. *Molecular Microbiology* **112**: 184-198.
